# Supplementary material for: The Pseudomonas aeruginosa substrate-binding protein Ttg2D functions as a general glycerophospholipid transporter across the periplasm
Source: Commun Biol. 2021 Apr 9;4:448. doi: 10.1038/s42003-021-01968-8 (PMC8035174; doi:10.1038/s42003-021-01968-8)
Supplement: Supplementary file 2 — Supplementary Information [file 42003_2021_1968_MOESM2_ESM.pdf]

SUPPLEMENTARY INFORMATION

The *Pseudomonas aeruginosa* substrate-binding protein Ttg2D functions as a general glycerophospholipid transporter across the periplasm

Daniel Yero,[1,2] Mireia Díaz-Lobo,[3] Lionel Costenaro,[1] Oscar Conchillo-Solé,[1] Adrià Mayo,[1] Mario Ferrer-Navarro,[1] Marta Vilaseca,[3] Isidre Gibert,[1,2]* Xavier Daura[1,4]*

[1]Institut de Biotecnologia i de Biomedicina (IBB), Universitat Autònoma de Barcelona (UAB), Barcelona, Spain; [2]Departament de Genètica i de Microbiologia, UAB, Barcelona, Spain; [3]Institute for Research in Biomedicine (IRB Barcelona), The Barcelona Institute of Science and Technology, Barcelona, Spain; [4]Catalan Institution for Research and Advanced Studies (ICREA), Barcelona, Spain.

*Corresponding Authors: Xavier Daura, Xavier.Daura@uab.cat and Isidre Gibert, Isidre.Gibert@uab.cat.

This document includes:

Supplementary Figures 1 – 13

Supplementary Tables 1 – 5

Supplementary Methods

Supplementary Discussion

Supplementary References

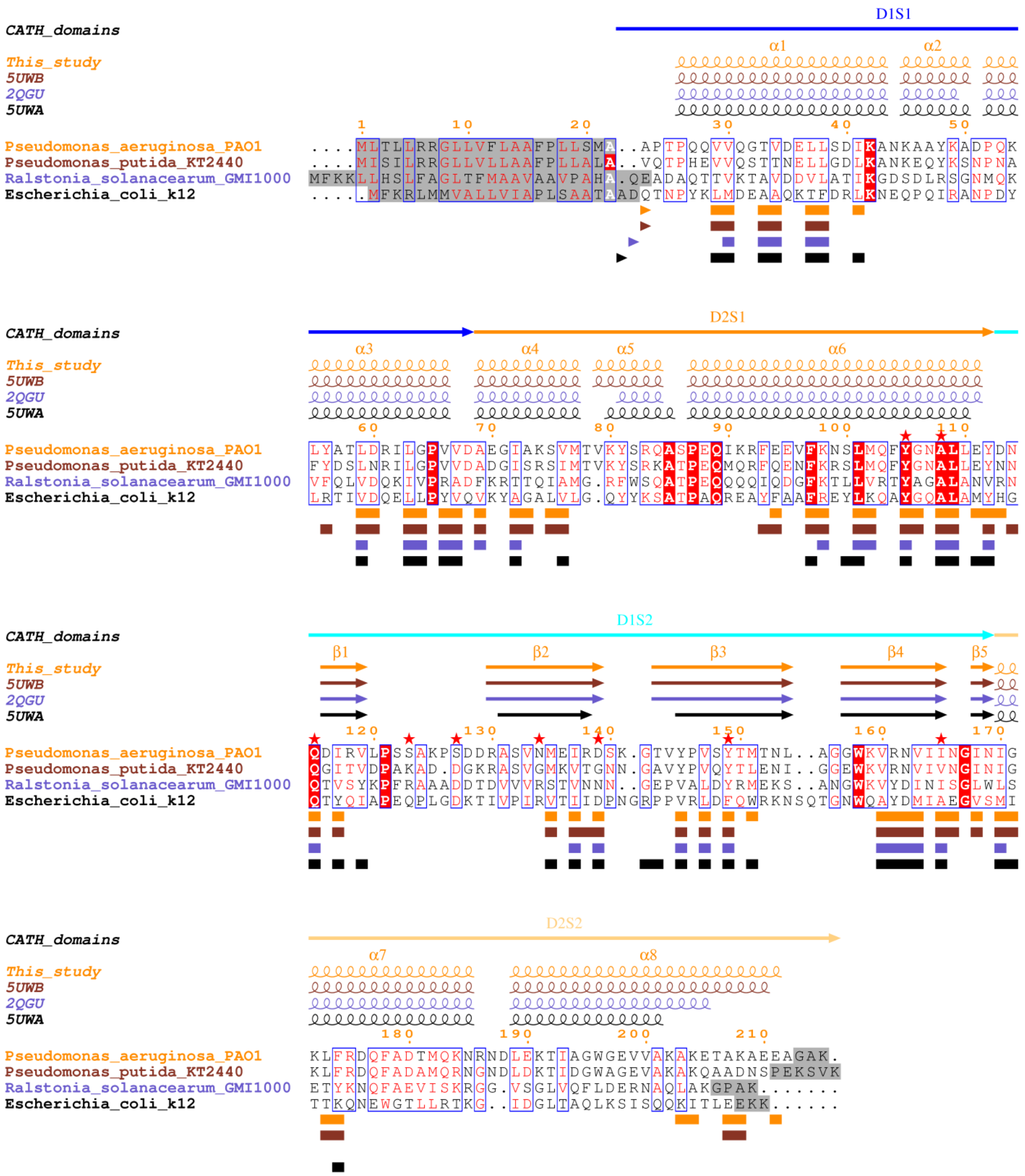

Supplementary Figure 1. Multiple-sequence alignment of MlaC family proteins with known 3D structure based on a structural alignment. CATH domains 1 (D1) and 2 (D2), each with segments 1 (S1) and 2 (S2), are shown above the sequences together with their secondary structure elements. Identical residues are highlighted in red, similar residues are in red font and similar regions are boxed in blue. Residues highlighted in grey are missing in the PDB entries. Sequence numbers correspond to the *P. aeruginosa* protein (Ttg2D$_{Pae}$). Red stars indicate residues annotated in the binding site of MlaC from *R. solanacearum* (2QGU). Below the sequences, triangles indicate the first amino acid of the mature protein (after cleavage of the signal peptide) and squares the residues forming the cavities.

A

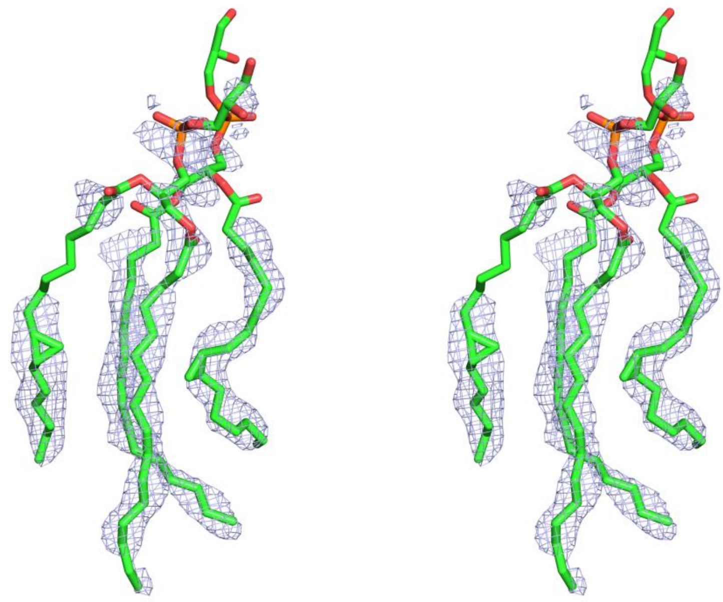

B

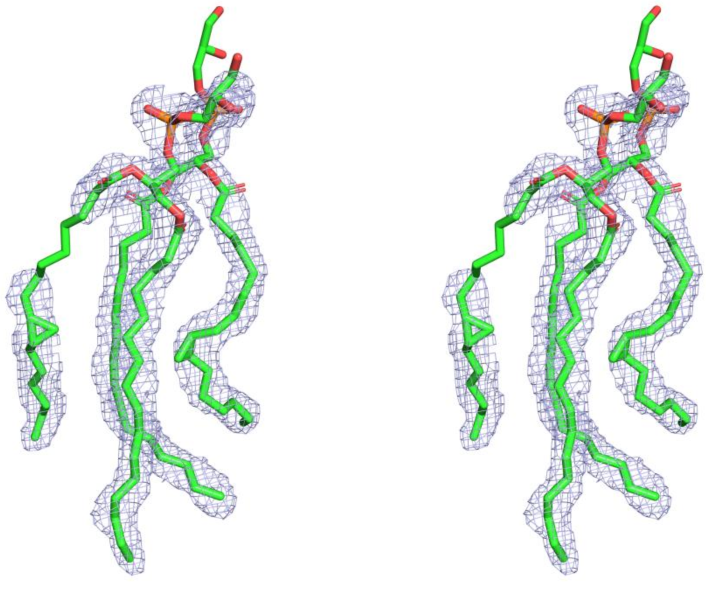

Supplementary Figure 2. (A) Unbiased 2mF_o - DF_c electron density map from AutoBuild (without any ligand added) and (B) final feature-enhanced electron density map (with modeled ligands) for the refined phospholipids. Both maps are contoured at 1σ (stereo view).

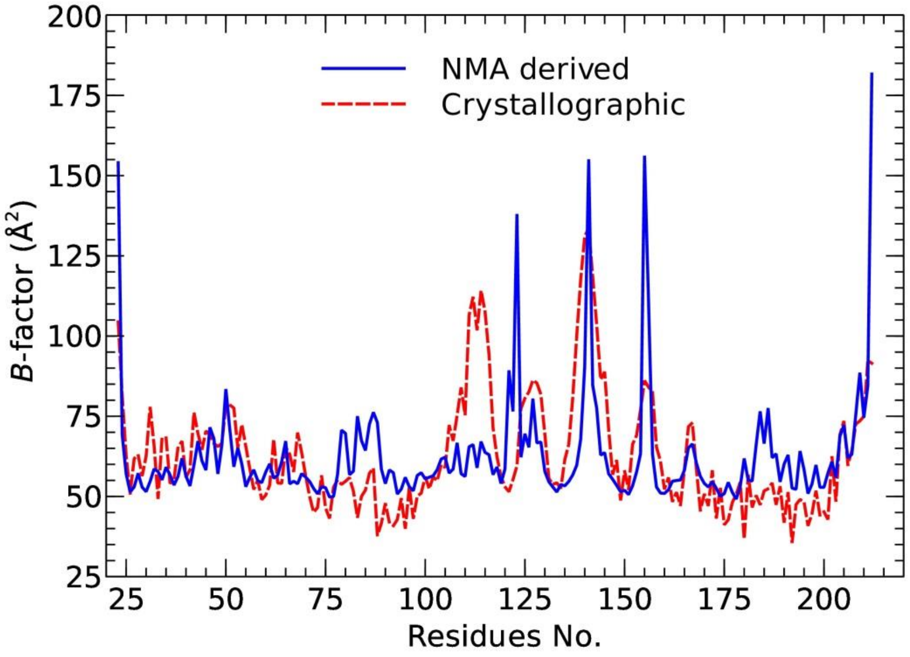

Supplementary Figure 3. Correlation between normal-mode-derived and crystallographic *B*-factors (Cα).

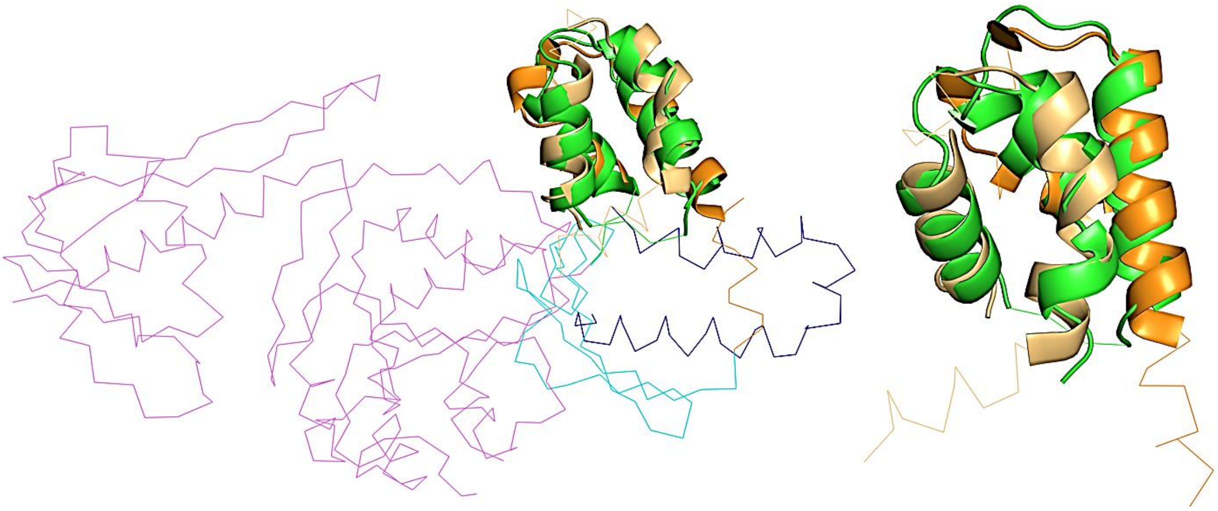

Supplementary Figure 4: Superposition of Ttg2D$_{Pae}$ with PDB 6UKS chain C, colored by domains. Ttg2D$_{Pae}$ with same colors as in Fig. 1 and Supplementary Fig. 1; 6UKS helical domain in green, other domains in magenta. Superposed residues according to DALI represented as cartoons, non-superposed as Cα-trace. Left panel: both proteins are shown. Right panel: only AAA (ATPases Associated with diverse cellular Activities) helical domain and Ttg2D$_{Pae}$ Domain D2 are represented.

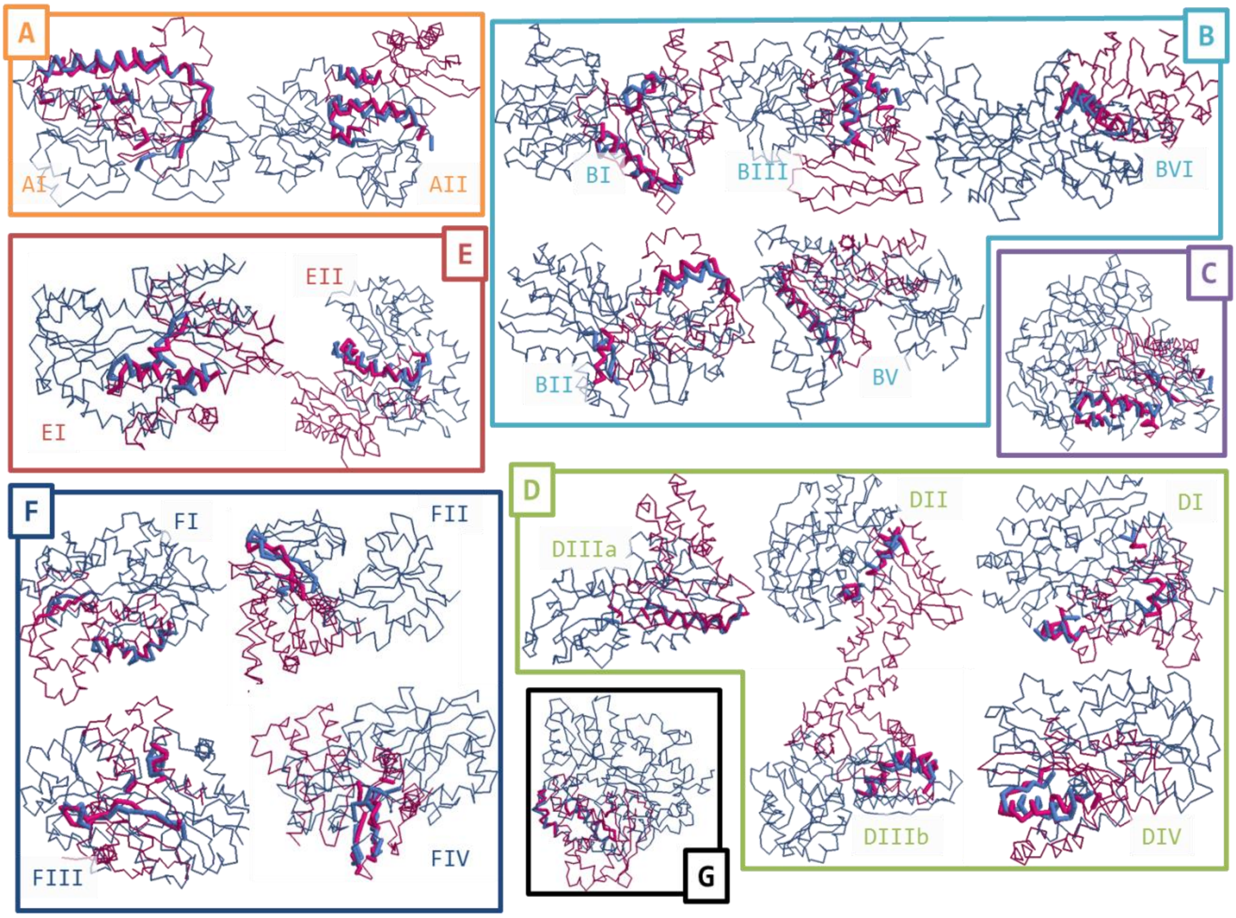

SBP PDB_Cluster	Aligned residues	RMSD Å	Fig. panel	SBP PDB_Cluster	Aligned residues	RMSD Å	Fig. panel
2prs_A-I	47	3,97	A	4exl_D-IIIa	22	2,29	D
2x4l_A-II	37	3,77	A	3cg1_D-IIIb	25	3,26	D
3s99_B-I	30	2,87	B	1o7t_D-IV	25	3,6	D
3om0_B-II	24	3,94	B	2cex_E-I	26	3,53	E
3sg0_B-III	29	3,88	B	2qpq_E-II	27	3,17	E
1jdn_B-IV	24	2,5	B	2x7p_F-I	28	3,73	F
3mq4_B-V	23	1,41	B	4ntl_F-II	19	3,73	F
2wok-C	40	3,76	C	1r9l_F-III	25	3,42	F
2z8d_D-I	24	3,55	D	1us4_F-IV	28	3,84	F
2qry_D-II	21	3,51	D	1y3p_G-A	31	3,75	G

Supplementary Figure 5: Superposition of Ttg2D$_{Pae}$ (purple structures) on a representative of each subcluster (gray structures) defined in the SBP classification by Scheepers GH et al.[16]. Superposed residues are shown in thick Cα-trace. The table lists the RMSD values and the number of aligned residues for each structural alignment.

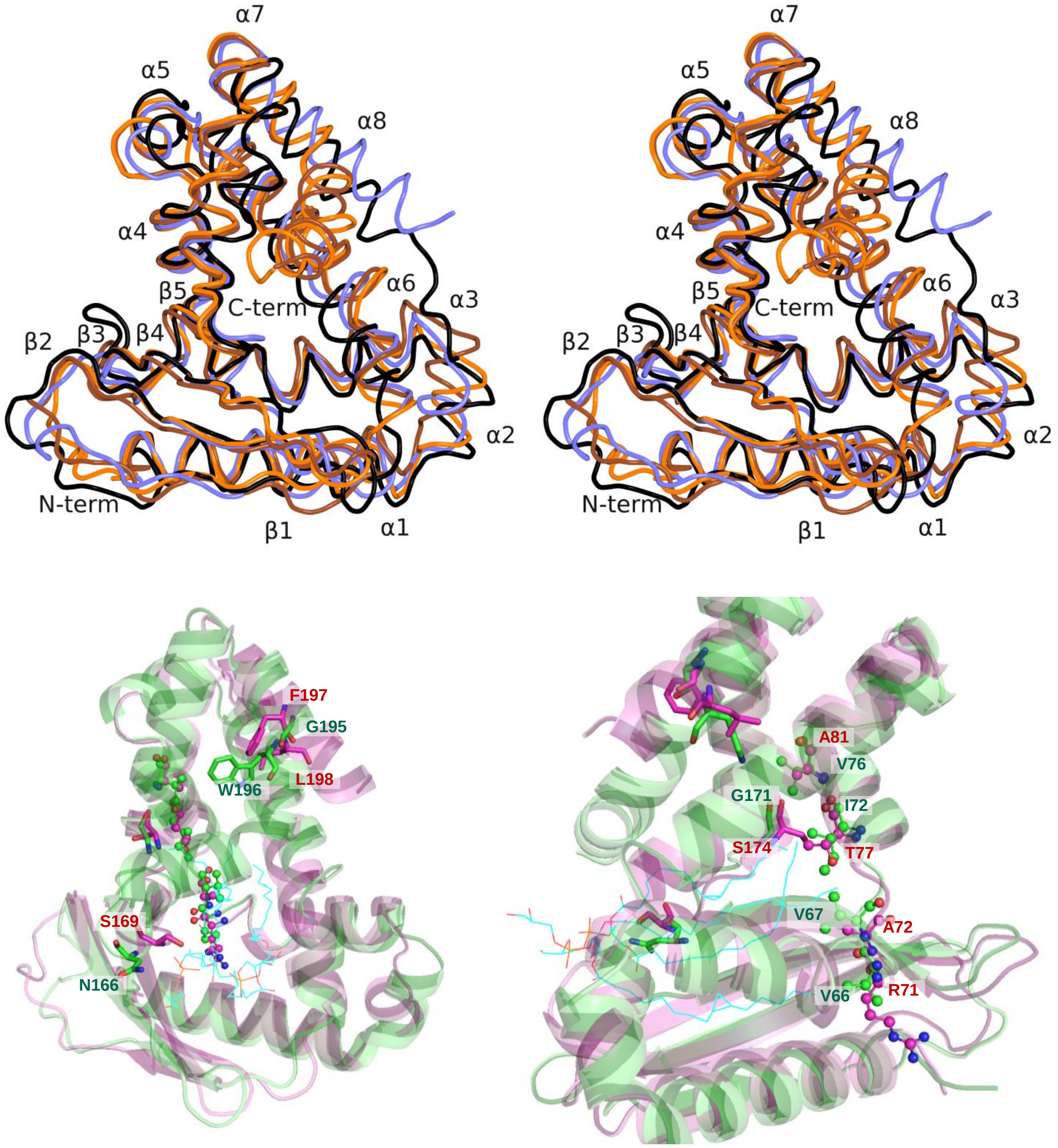

Supplementary Figure 6. Superposition of the structure of Ttg2D from *P. aeruginosa* with known orthologous protein structures. Upper panels show superposition of Ttg2D crystal structures from *P. aeruginosa* (this study, PDB entry 6HSY), *P. putida* (5UWB), *R. solanacearum* (2QGU) and *E. coli* (5UWA) (stereo view). Same color coding as in Supplementary Fig. 1. Lower panels show superposition of the structures of Ttg2D from *P. aeruginosa* (green) and *R. solanacearum* (pink) in two different views. Residues that distinguish the group of proteins that we predict to bind two diacyl lipids are indicated with residue letter and number. Besides G195 and W196, side-chain orientation and hydrophobicity of the other indicated residues in these regions could be also contributing to a tighter binding of the two diacyl phospholipids inside the ligand cavity. Residues in region 65-83 are represented as *ball-and-stick and* residues in region 154-198 as *sticks*.

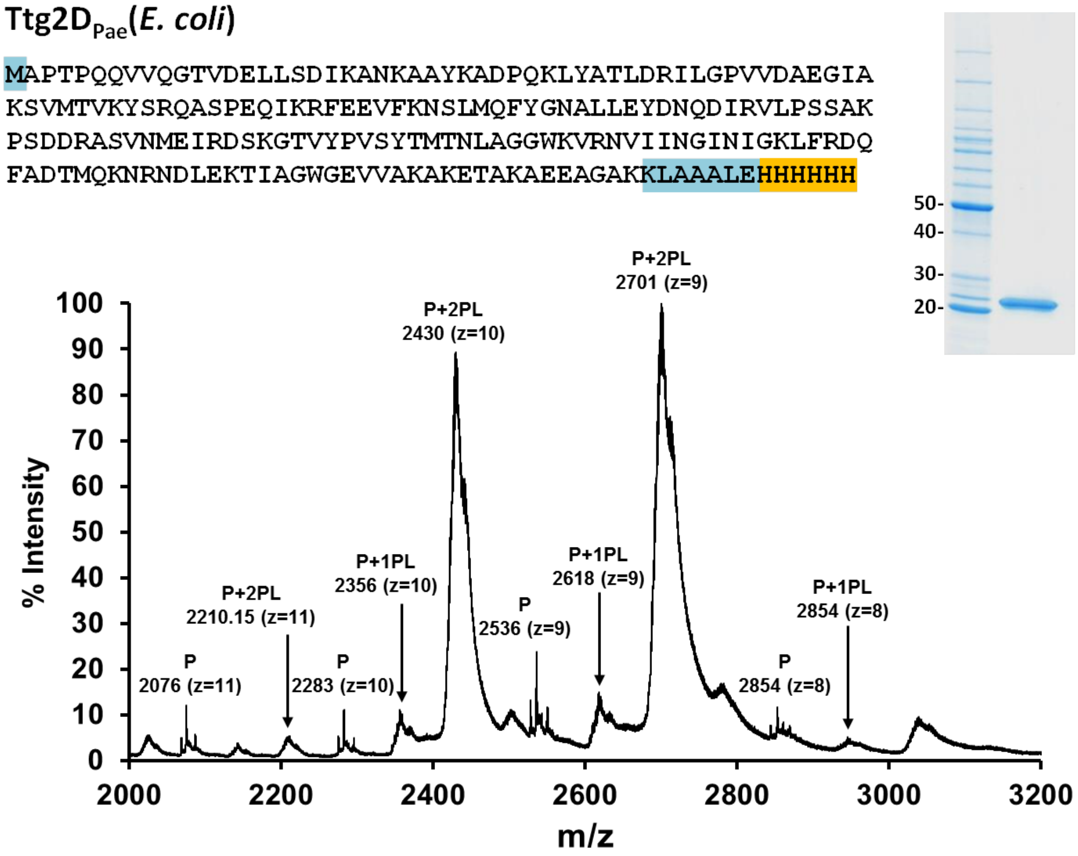

Supplementary Figure 7. Recombinant Ttg2D$_{Pae}$, produced in *E. coli*. Zoom of the 2000-3000 m/z range of the mass spectrum of Ttg2D$_{Pae}$, produced in *E. coli* under native conditions in positive ion mode (The full mass spectrum is shown in Fig. 3A). The most represented species are complexes of the protein (P) containing either one (1PL, deconvoluted MW=23551 Da) or two (2PL, deconvoluted MW=24296) phospholipid molecules. Above the spectrum, the inferred amino acid sequence for the recombinant protein is displayed, highlighting the 6xHis tail in yellow and other added amino acids from pET vector in blue. The SDS-PAGE shows the purified recombinant protein (first column: MW marker in kDa). The theoretical monoisotopic MW of the recombinant protein that has lost the first methionine is 22836.78 Da.

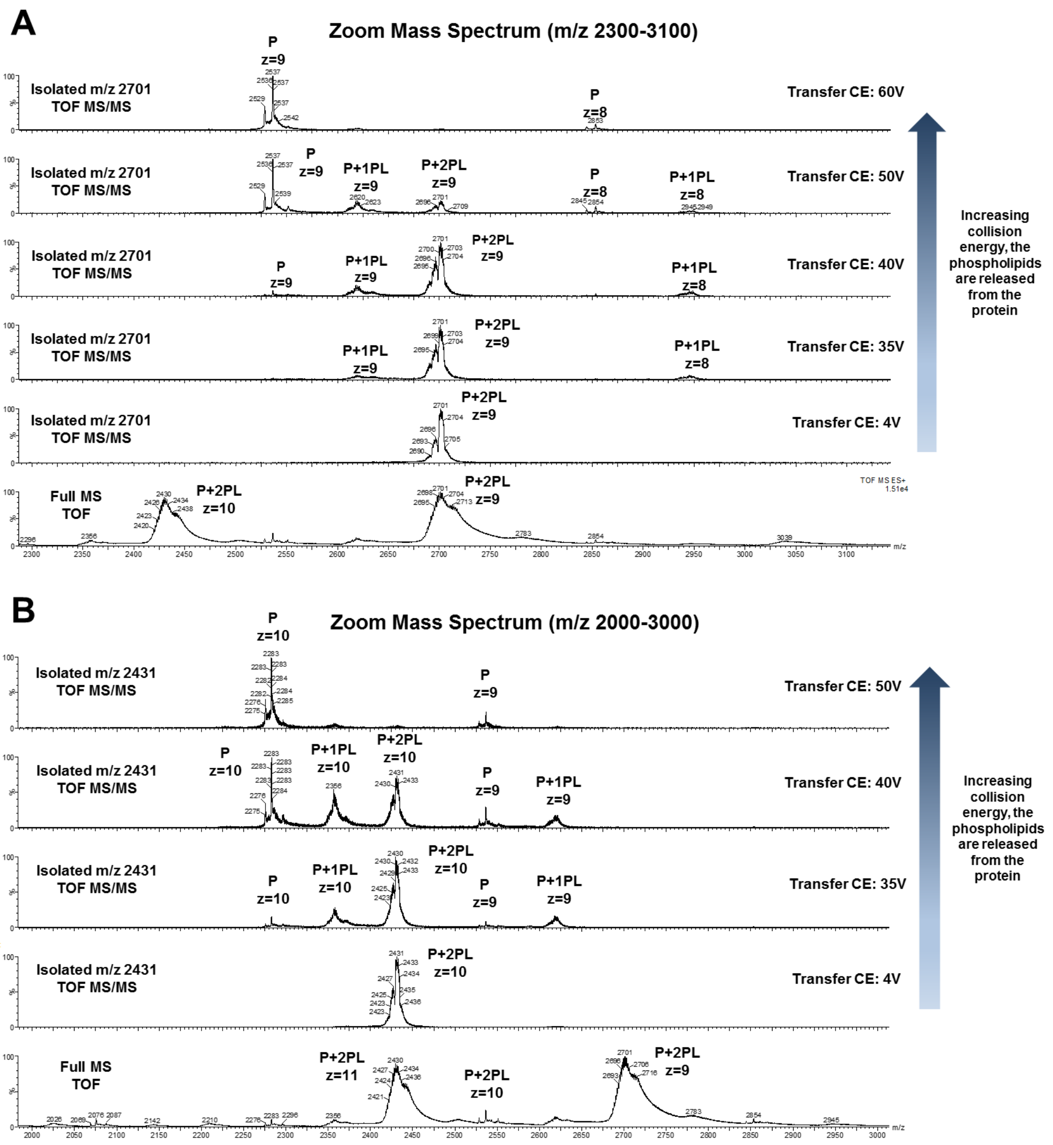

Supplementary Figure 8. Complexes formed by protein $Ttg2D_{Pae}$ produced in *E. coli* (P) and phospholipids (PL). The dissociation of the $(P+2PL)^{9+}$ complex (m/z 2700 peak) yields the complementary $(P+1PL)^{8+}$ and $(PL)^{+}$ product ion pair, but also the $(P+1PL)^{9+}$ with concurrent loss of neutral PL. In addition, the $(P+1PL)^{9+}$ complex dissociates into $(P)^{8+}$ and $(PL)^{+}$ ions, furthermore, the $(P)^{9+}$ apo-protein with concurrent loss of neutral PL was detected as well. MS/MS of the $(P+2PL)^{10+}$ complex (m/z 2431 peak) shows identical behavior. (A) Zoom of the 2300–3100 m/z range of the fragment ion mass spectra of isolated ion m/z=2700 (z=9) at varying transfer collision energies (CE). Isolation and dissociation of the species P+2PL at CE greater than 30V have allowed the detection of species with one ligand (P+1PL). However, at CE of 50V there still exists a population of fully charged species were the 2PL could correspond to CL-like molecules. Increasing the CE results in complete release of the phospholipids. The mass of the bound molecules can then be calculated from the mass difference between the bound and unbound peaks. (B) Zoom of the 2000-3000 m/z mass spectra range of fragmentations of isolated ion m/z=2430 (z=10) at varying transfer collision energies (CE). Increasing the CE results in partial release of the bound ligands. Note that 2PL could be a mixture of populations with two different phospholipids or one cardiolipin molecule.

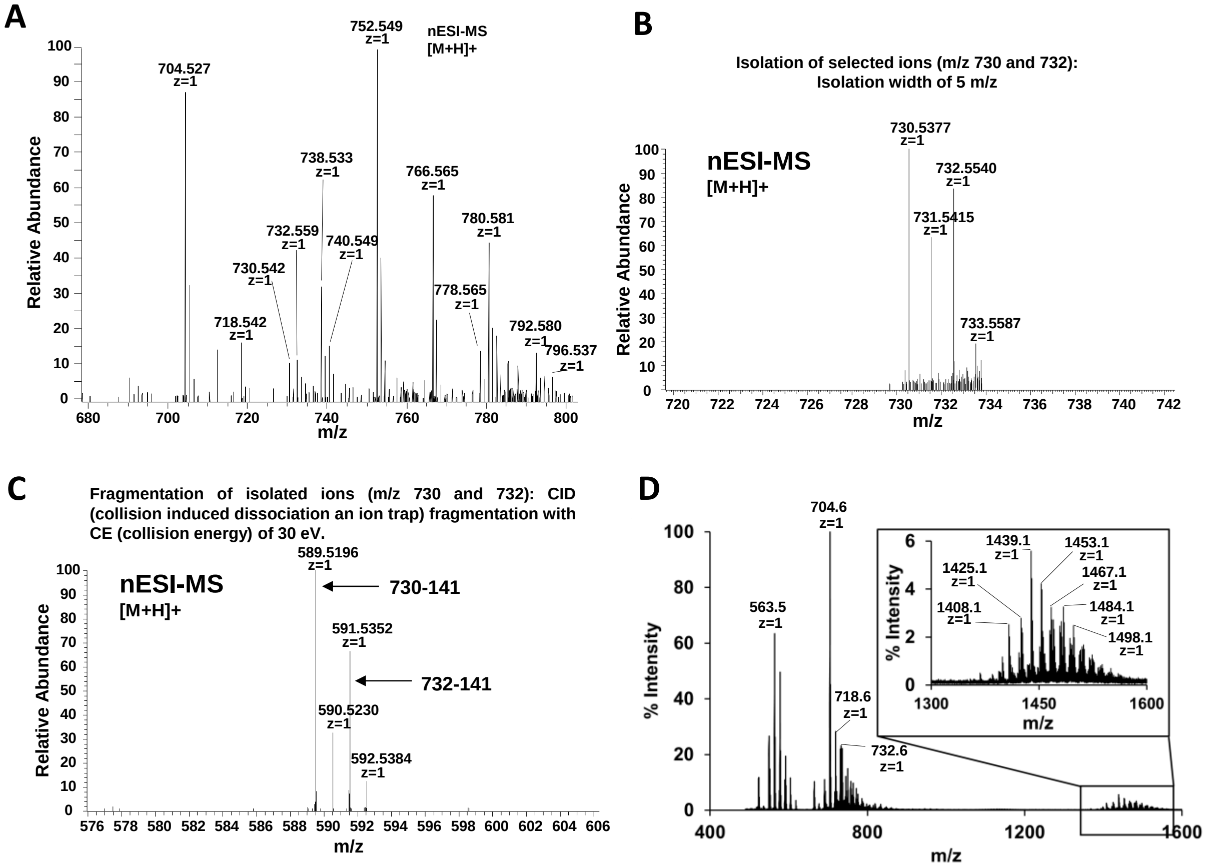

Supplementary Figure 9. Lipid species in recombinant Ttg2D$_{Pae}$ produced in *E. coli* detected by nano-electrospray ionization mass spectrometry (nESI-MS) in positive ion mode at high resolution (100 k) and accuracy (<3ppm) (A). A detailed list of detected peaks is shown in Supplementary Table 2. Selected ions (m/z 730, z=1 and m/z 732, z=1) were isolated (B) and later fragmented in the gas phase to prove their lipid nature (C) observing the loss of the head of the phospholipid class phosphoethanolamine (-141 Da). Lipid and cardiolipin species in recombinant Ttg2D$_{Pae}$ produced in *E. coli* detected by LC-MS under denaturing conditions in positive mode (D).

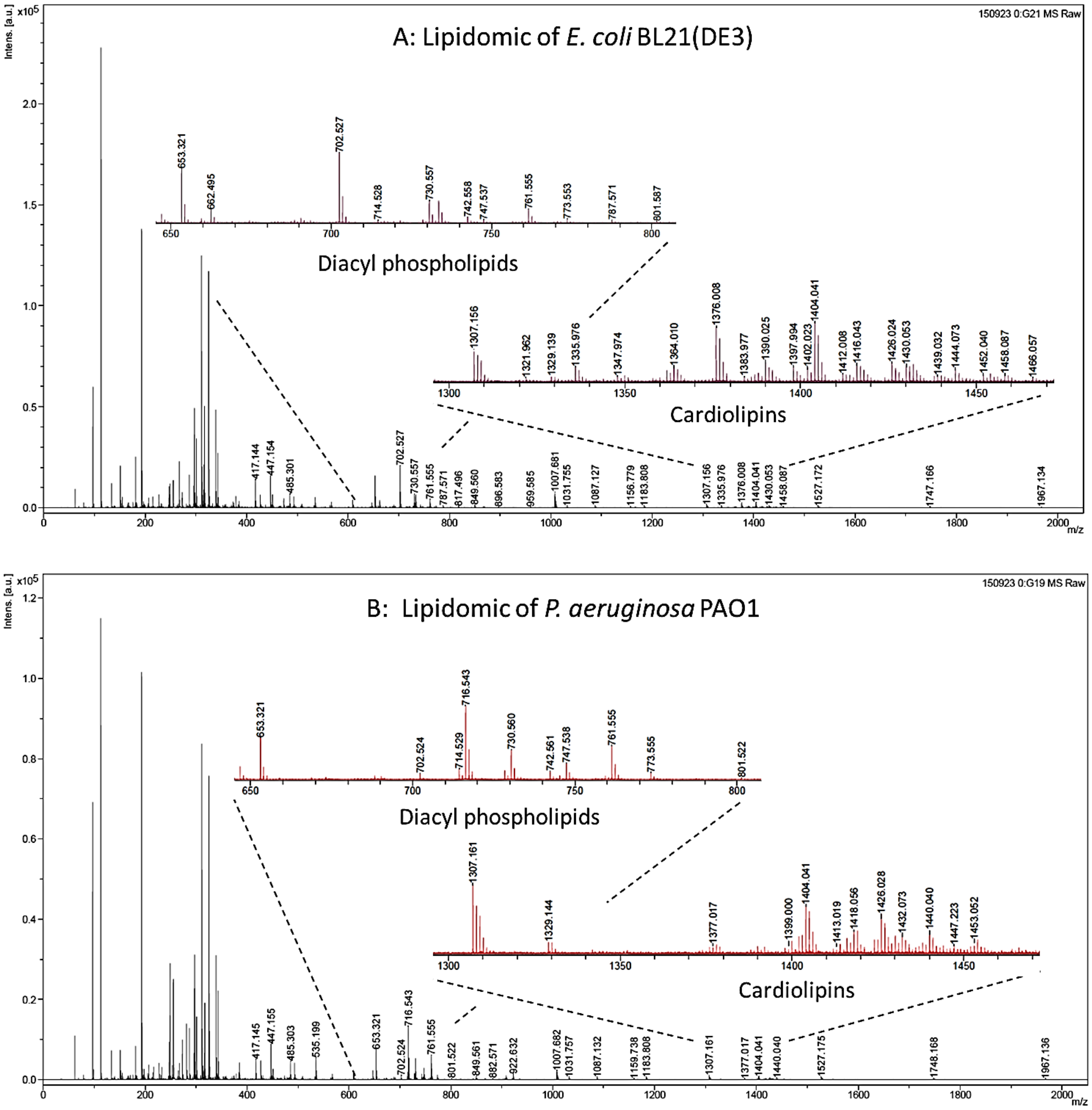

Supplementary Figure 10. Negative MALDI-TOF/MS lipid profiles of the major membrane phospholipids in *E. coli* BL21(DE3) (A) and *P. aeruginosa* PAO1 (B) acquired using 9-AA as matrix.

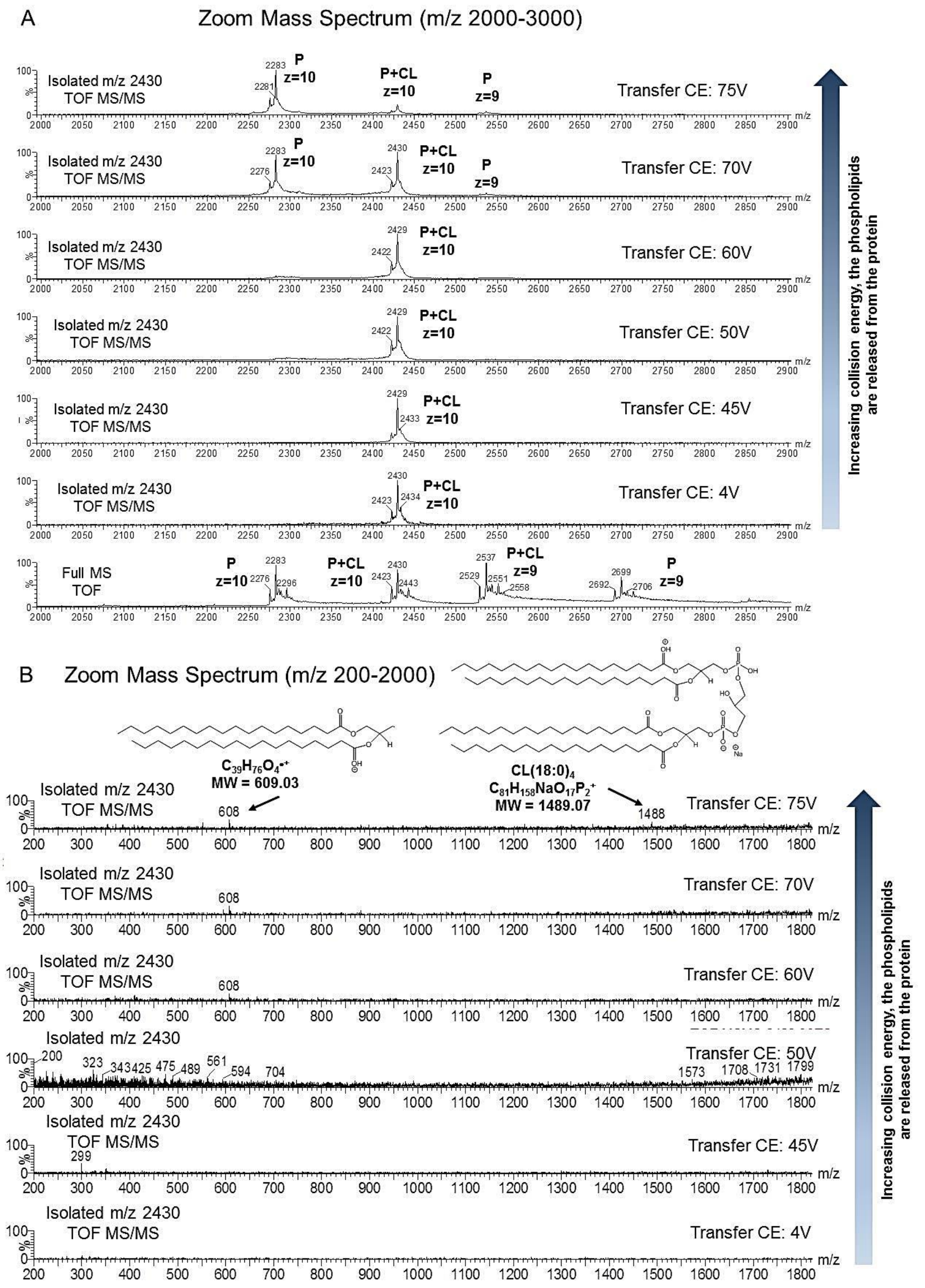

Supplementary Figure 11. Fragmentations of isolated ion m/z=2430 (z=10) at varying transfer collision energies (CE) from the ligand–binding assays to study the interaction *in vitro* between delipidated Ttg2D$_{Pae}$ (P) and cardiolipin (CL). Dissociation of the $(P+CL)^{10+}$ complex yields the complementary $(P)^{9+}$ and $(CL)^{+}$ product ion pair as one dissociation pathway, and the $(P)^{10+}$ protein with loss of neutral CL as another pathway. (A) Zoom of the 2000–3000 m/z mass spectra range. Increasing the CE results in partial release of the bound cardiolipin. (B) Zoom of the 200–2000 m/z mass spectra range. At the transfer CE required for cardiolipin release this molecule is easily fragmented. Fragmentation of cardiolipin usually occurs at the phosphate ester bonds to glycerol or at the ester bond of the fatty acids[17].

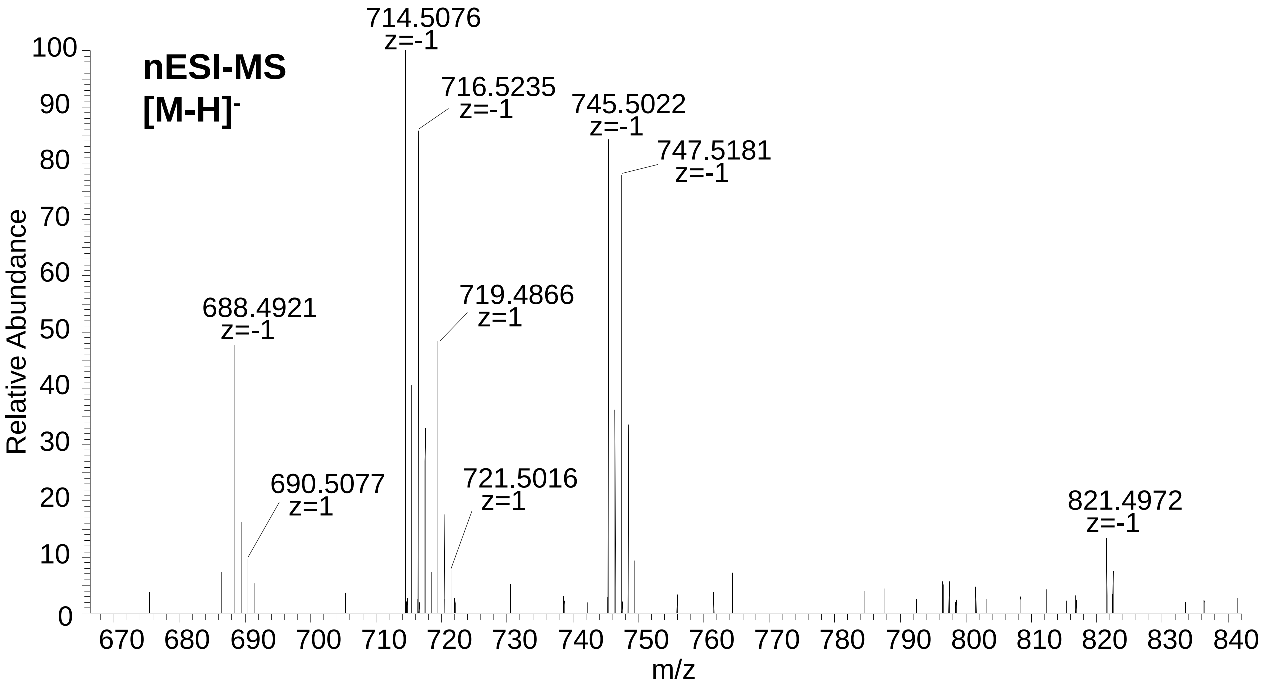

Supplementary Figure 12. Lipid species in Ttg2D$_{Pae}$ produced in *P. aeruginosa* detected by nanoelectrospray ionization mass spectrometry (nESI-MS) in negative mode at high resolution (100 k) and accuracy (<3ppm). A detailed list of detected peaks in negative mode is shown in Supplementary Table 3.

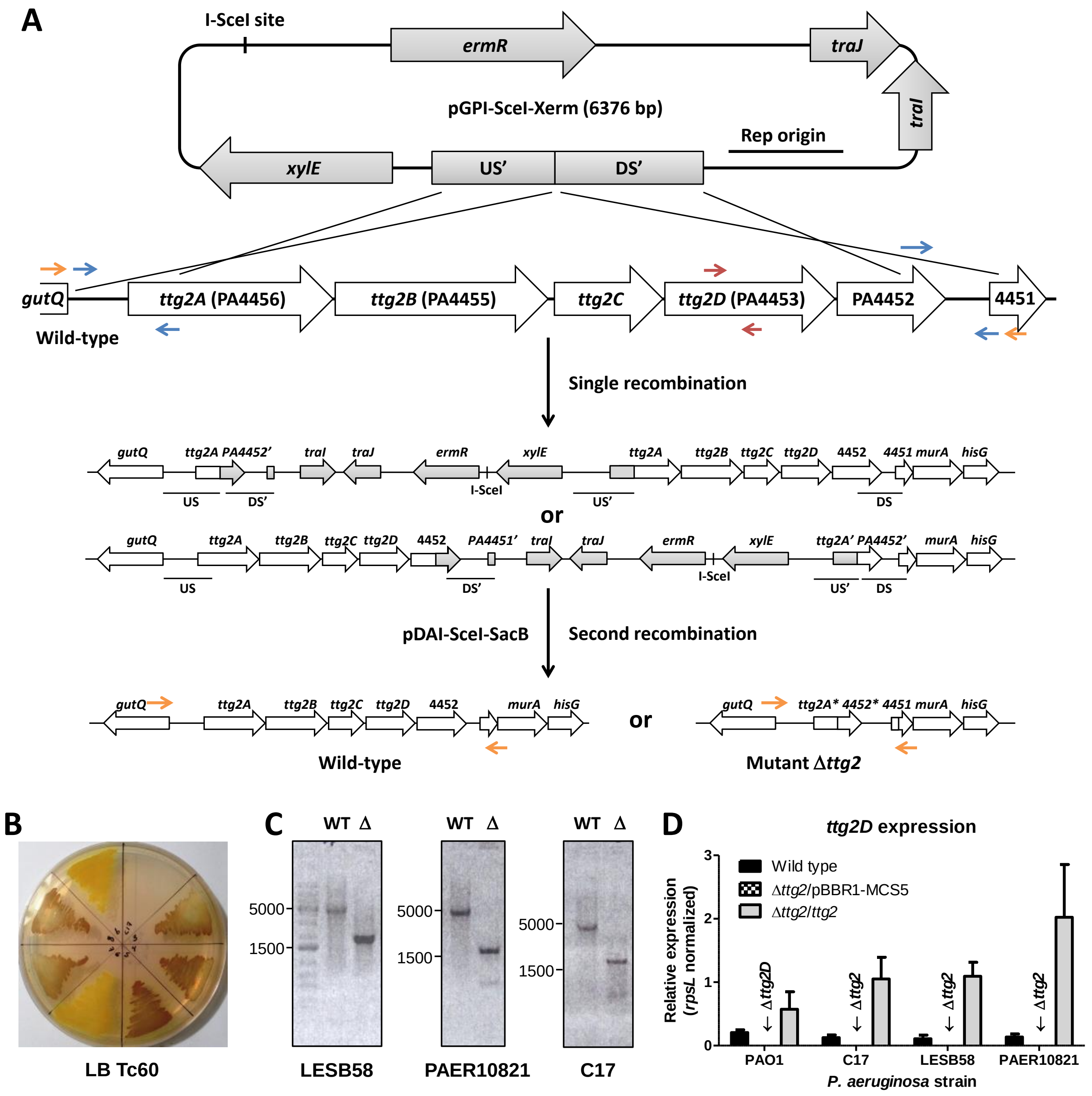

Supplementary Figure 13: Generation of *ttg2* mutants in MDR *P. aeruginosa* strains using a modified pGPI-SceI/pDAI-SceI-SacB system. (A) Schematic representation of the construction of the *ttg2* knockout mutant (genes and locus tags as in PAO1). See supplementary methods for details. Primer pairs for amplification of the upstream (US) and downstream (DS) fragments for recombination events are represented as blue arrows. The second recombination event generates the desired mutant or results in reversion to wild type (WT). (B) Screening for the spontaneous resolution of cointegrates in *P. aeruginosa* strain C17 in the presence of pyrocatechol. In the presence of this compound, colonies expressing 2,3-catechol-dioxygenase encoded by *xylE* turn yellow, while *P. aeruginosa xylE* negative colonies turn dark brown. The picture shows a selective agar plate containing representative colonies after spraying with pyrocatechol. (C) Screening by PCR to confirm the deletion using external primers represented as orange arrows in panel A. Expected amplicon sizes in the WT and in the mutant (Δ) are 4755 bp and 1849 bp respectively. (D) Real-time PCR (qPCR) of cDNA amplified from WT and *ttg2* mutant and complemented strain (with plasmid pBBR1MCS-5-*ttg2*) using *ttg2D* internal primers (represented as red arrows in panel A). Expression levels were normalized to the *rpsL* levels.

Supplementary Table 1. Structural alignment parameters and cavity volumes of Ttg2D proteins.

Species (strain)	PDB code	RMSD in Å (No. Cα) ‡	Identity‡	Similarity‡	Volume† in Å³	No. cavity's aa/atoms†	Ref.
Pseudomonas aeruginosa (PAO1)	6HSY	0.0 (190)	100%	100%	2979	55/185	This study
Pseudomonas putida (KT2440)	5UWB	1.6 (188)	63%	78%	2510	52/166	[1]
					2337	49/153	
Ralstonia solanacearum (GMI1000)	2QGU	2.3 (180)	25%	48%	1332	31/101	N/A
Escherichia coli (K12)	5UWA	3.1 (185)	17%	39%	1444	43/118	[1]
					1369	43/111	

‡Structural alignment parameters obtained from POSA web server for the PDB sequences.
†Molecular-surface volume (without H atoms) and the number of amino acid residues (aa) and atoms in the cavity for protein chain (chains A and B in *P. putida* and *E. coli*).

Supplementary Table 2. PL released species detected by mass spectrometry from lipid-protein complexes of recombinant Ttg2D$_{Pae}$ produced in the cytoplasm of *E. coli*.

PL class‡	Monoisotopic (exact mass)	nESI-MS mode	Possible combination of fatty acids (PL species)†	Lipidome analyses of *E. coli* (negative mode)#
PE	663.480	Positive/Negative	16:0/14:0	Yes
PG	694.483	Negative	16:0/14:0	
**PE**	**703.499**	**Positive/Negative**	**16:0/cy17:0**	**Yes**
PE	717.535	Positive	16:0/18:1	
PG	720.499	Negative	14:0/18:1<br>16:0/16:1<br>15:0/cy17:0	
PG	722.515	Negative	16:0/16:0	
PE	729.535	Positive	16:1/cy19:0	
**PE**	**731.552**	**Positive**	**17:0/18:1**<br>**16:0/cy19:0**<br>**18:0/cy17:0**	**Yes**
**PG**	**734.513**	**Negative**	**16:0/cy17:0**	**Yes**
PE/PC	737.525	Positive	(36:5)/(33:5)	
PE/PC	739.542	Positive	(36:4)/(33:4)	
**PG**	**748.529**	**Negative**	**16:0/18:1**	**Yes**
**PE/PC**	**751.541**	**Positive**	**(37:5)/(34:5)**	
PE/PC	753.520	Positive	(37:4)/(34:4)	
PG	762.545	Negative	16:0/cy19:0	Yes
PE/PC	765.557	Positive	(38:5)/(35:5)	
PG	770.546	Positive	(36:4)	
PG	774.547	Negative	18:1/18:1	Yes
PC	777.557	Positive	17:0/18:0	
PG	788.563	Negative	18:1/cy19:0	Yes
PS	791.573	Positive	18:0/18:0	
PC	793.552	Positive	(37:5)	
PS	795.530	Positive	(37:5)	

‡ The most abundant species in the mass spectra are indicated in bold. Peaks for which a clear PL species or class could not be assigned are not listed. Phosphoethanolamine (PE); Phosphatidylglycerol (PG); Phosphatidylcholine (PC); Phosphatidylserine (PS).

† Phospholipid species (PL) based on the most abundant fatty acids and PL reported in *E. coli*[2, 3]. Numbers associated to each species indicate the number of carbon atoms and double bonds, respectively, for each fatty acid side chain. Fatty acid cyclopropylation (cy) is equivalent to unsaturation in terms of molecular mass. When it was not possible to assign the fatty acid species, the total number of carbon atoms and double bonds is shown in parentheses.

# Lipidome analyses made for this work are shown in Supplementary Fig. 10. Column indicates whether PL was detected in these analyses.

Supplementary Table 3. Phosphoethanolamine and phosphatidylglycerol released species detected by mass spectrometry from lipid-protein complexes of Ttg2D$_{Pae}$ produced in the periplasm of *P. aeruginosa*.

PL class‡	Monoisotopic (exact mass)	nESI-MS mode	Possible combination of fatty acids (PL species)†	Lipidome analyses of *P. aeruginosa* (negative mode)#
PE	689.499	Negative	16:0/16:1	
PE	691.515	Negative	16:0/16:0	
**PE**	**715.515**	**Negative**	**16:1/18:1**	**Yes**
**PE**	**717.530**	**Negative**	**16:0/18:1**	**Yes**
PG	720.494	Negative	16:0/16:1	
PG	722.509	Negative	16:0/16:0	
**PG**	**746.509**	**Negative**	**16:1/18:1**	**Yes**
**PG**	**748.525**	**Negative**	**16:0/18:1**	**Yes**

‡ The most abundant species in the mass spectra are indicated in bold. Peaks for which a clear PL species or class could not be assigned are not listed. Phosphoethanolamine (PE); Phosphatidylglycerol (PG).
† Phospholipid species (PL) based on the most abundant fatty acids and PL reported in *P. aeruginosa*[4]. Numbers associated to each species indicate the number of carbon atoms and double bonds, respectively, for each fatty acid side chain.
# Lipidome analyses made for this work are shown in Supplementary Fig. 10. Column indicates whether PL was detected in these analyses.

Supplementary Table 4. Antibiotic susceptibility profile of *P. aeruginosa* mutants of the Ttg2/VacJ system and complemented strains for Δ*ttg2*D.

Antibiotic	MIC† in µg/ml							
	PAO1	Δ*ttg2*D	Δ*ttg2*C	Δ*ttg2*B	Δ*ttg2*A	Δ*vacJ*	Δ*ttg2D*/ pBBR1-MCS-5-*ttg2*	Δ*ttg2D*/ pBBR1-pBAD-*ttg2*D
Polypeptides								
Colistin	0.5	0.0625*	0.0625*	0.0156*	0.0312*	0.0625*	0.5	0.25
Polymyxin B	2	0.75*	1	1	1	1	4	ND
Fluoroquinolones								
Ciprofloxacin	1	0.0625*	0.0625*	0.0312*	0.0312*	0.0625*	0.125*	0.125*
Levofloxacin	4	0.125*	0.125*	0.0625*	0.0625*	0.125*	0.5*	0.25*
Ofloxacin	8	0.25*	0.25*	0.25*	0.5*	0.5*	1*	0.5*
Norfloxacin	4	0.25*	0.25*	0.25*	0.25*	0.25*	0.5*	0.5*
Tetracyclines								
Minocycline	32	2*	2*	2*	2*	2*	8*	8*
Tigecycline	32	4*	4*	4*	4*	4*	16	16
Chloramphenicol								
Chloramphenicol	>256	32*	32*	16*	32*	32*	64*	64*
Aminoglycosides								
Tobramycin	0,25	0.5	0.5	0.5	0.5	0.5	2	2
Amikacin	2	4	4	2	4	4	4	8
Streptomycin	8	32*	64*	32*	32*	32*	64	64
Carbapenems (beta-lactam)								
Imipenem	4	1*	1*	2	1*	1*	1*	1*
Meropenem	1	0.5	0.5	1	0.5	0.5	1	0.25*
Cephalosporins (beta-lactam)								
Ceftazidime	1	2	4*	2	2	2	2	2
Penicillins (beta-lactam)								
Piperacillin	2	4*	8*	8*	16*	8*	4	8*
Ticarcillin	16	32	32	32	32	32	16	32

†Minimum inhibitory concentration (MIC) determined by the broth microdilution method except for polymyxin B that was determined by Etest (ND: not determined). MICs were confirmed by two or three independent replicates and MIC differences greater than 2-fold with respect to the wild type strain were considered significant (indicated with an asterisk). Transposon mutants of *P. aeruginosa* PAO1 and complemented strains are described in Supplementary Table 5.

Supplementary Table 5: Bacterial strains, plasmids and oligonucleotides used to study the role of the *ttg2* operon in *Pseudomonas aeruginosa*.

Strain name	Genotype	Description	Reference
Pseudomonas aeruginosa			
MPAO1[‡]	Wild type	Subline of PAO1. Strain lacking a transposon insertion. PAO1 is the standard laboratory and genetic reference strain.	[5]
PAO1Δ*ttg2D*	*ttg2D*-D04::IS*phoA*/hah (PW8497)[†]	Ttg2D (PA4453) mutant; Tet^r	[5, 6]
PAO1Δ*ttg2C*	*ttg2C*-A12::IS*phoA*/hah (PW8498)[†]	Ttg2C (PA4454) mutant; Tet^r	[5, 6]
PAO1Δ*ttg2B*	*ttg2B*-A07::IS*lacZ*/hah (PW8500)[†]	Ttg2B (PA4455) mutant; Tet^r	[5, 6]
PAO1Δ*ttg2A*	*ttg2A*-B04::IS*phoA*/hah (PW8503)[†]	Ttg2A (PA4456) mutant; Tet^r	[5, 6]
PAO1Δ*vacJ*	*vacJ*-H08::IS*phoA*/hah (PW5688)[†]	VacJ (PA2800) mutant; Tet^r	[5, 6]
LESB58 [§]	Wild type	A highly virulent epidemic strain (LES) first identified in the Liverpool CF clinic center, β-lactam resistant.	[7, 8]
LESB58Δ*ttg2*	Δ*ttg2ABCDE*	LESB58 carrying a deletion in *ttg2* operon.	This study
C17 [¶]	Wild type	Clinical isolate from rectal swabs.	This study
C17Δ*ttg2*	Δ*ttg2ABCDE*	C17 carrying a deletion in *ttg2* operon.	This study
PAER-10821 [¶]	Wild type	Human clinical isolate.	This study
PAER-10821 Δ*ttg2*	Δ*ttg2ABCDE*	PAR10821 carrying a deletion in *ttg2* operon.	This study
Escherichia coli			
DH5α	F$^-$ Φ80*lacZ*ΔM15 Δ(*lacZYA-argF*) U169 *recA1 endA1 hsdR17*(r_K^- m_K^+) *phoA supE44 thi-1 gyrA96 relA1*λ$^-$	For cloning purposes.	[9]
HY327	Δ(*lac pro*) *argE*(*Am*) *recA56 rif*^{R} *nalA* λ *pir*	Expresses the λ Pir protein required for cloning and propagation of plasmids with the R6K origin of replication.	[10]
BL21(DE3)	F$^–$ *ompT gal dcm lon hsdS$_B$*($r_B^–m_B^–$) λ(DE3 [*lacI lacUV5-T7p07 ind1 sam7 nin5*]) [*malB*$^+$]$_{K-12}$(λ^S)	Host for recombinant protein expression from plasmids containing T7 promoter.	Novagen
Plasmid		Description	Reference
pGPI-SceI-XCm		Mobilizable suicide vector; carries the R6Kγ origin of replication, the I-SceI recognition site and a *xylE* reporter gene, Cm^r, Tp^r	[11]
pGPI-SceI-XErm		Modified pGPI-SceI-XCm vector, Erm^r, Tp^r	This study
pΔ*ttg2*-US'		pGPI-SceI-XErm with a 633-bp *Xba*I/*Xho*I insert of PAO1 containing the flanking region upstream of *ttg2*	This study
pΔ*ttg2*-US'DS'		pΔ*ttg2*-US with a 818-bp *Xho*I/*EcoR*I insert of PAO1 containing the flanking region downstream of *ttg2*	This study

pRK2013		RK2-derived helper plasmid carrying the *tra* and *mob* genes for mobilization of plasmids containing *oriT*, Kan^r	12
pDAI-SceI-SacB		Mobilizable broad host range plasmid; carries the gene for the I-SceI homing endonuclease and the *sacB* gene, Tet^r	11, 13
pBBR1MCS-5		Broad-host-range cloning vector used for complementation, low copy, Gm^r	14
pBBR1MCS-5-*ttg*2		pBBR1MCS-5 with the *ttg2* operon (PA4456-4452) inserted between sites *Xba*I and *Kpn*I (opposite orientation of lacZ promoter transcription), Gm^r	This study
pBBR1MCS-6		Modified pBBR1MCS-5 vector, Erm^r	This study
pBBR1MCS-6-*ttg*2		pBBR1MCS-6 with the *ttg2* operon (PA4456-4452) inserted between sites *Xba*I and *Kpn*I, Erm^r	This study
pBAD18-Cm		Expression vector containing the arabinose pBAD promoter and *araC*, Cm^r	15
pBBR1-pBAD-Gm		pBBR1MCS-5 containing the arabinose pBAD promoter and *araC* from pBAD18-Cm, Gm^r	This study
pBBR1-pBAD-*ttg2D*		pBBR1-pBAD-Gm with the *ttg2D* (PA4453) CDS inserted between sites *Nhe*I and *Hind*III, Gm^r	This study
pBBR1-pBAD-*ttg2DHis*		pBBR1-pBAD-Gm with the *ttg2D* (PA4453) CDS (encoding for six C-terminal histidines) inserted between sites *Nhe*I and *Hind*III, Gm^r	This study
pET28b		Bacterial expression vector with T7-lacO promoter, hexa His tag (Nterm and Cterm) with Thrombin cleavage (N terminal on backbone), Kan^r	Novagen
Primer name	Sequence 5’ to 3’	Description	Reference
US’-ttg2-U	GACGGAATTCTGGGCGGAATGGATGAAATC	Upstream forward primer to create pΔ*ttg2*-US’, *EcoR*I	This study
US’-ttg2-L	TATGCTAGCTCAGCCGCAGCAACGTGGTC	Upstream reverse primer to create pΔ*ttg2*-US’, *Nhe*I	This study
DS’-ttg2-U	CCAGCTAGCGCAGCCTTCTGGAGATCCTG	Downstream forward primer to create pΔ*ttg2*-US’DS’, *Nhe*I	This study
DS’-ttg2-L	GACAGATCTCCGCGGATATGCAGGTCGAC	Downstream reverse primer to create pΔ*ttg2*-US’DS’, *Bgl*II	This study
Ext-ttg2-U	GAGGTCGGGGCCAGGTTCAGTG	Forward primer outside the deleted region for mutant verification	This study
Ext-ttg2-L	CCTTAGCCTTGATGTAGCCGCCTTC	Reverse primer outside the deleted region for mutant verification	This study
Int-ttg2D-U	CAAGGCCGATCCGCAAAAGCTC	Forward primer for RT-PCR (amplicon size of 215 bp)	This study
Int-ttg2D-L	GCACGCGGATGTCCTGGTTGTC	Reverse primer for RT-PCR (amplicon size of 215 bp)	This study
ttg2compF	GTGTCTAGAGCGGAATGGATGAAATCG	Forward primer for cloning operon *ttg2* (PA4456-PA4452) into pBBR1MCS-5, *Xba*I	This study
ttg2compR	ATAGGTACCTTAACGTCTTCGGCCTGC	Reverse primer for cloning operon *ttg2* (PA4456-PA4452) into pBBR1MCS-5, *Kpn*I	This study

Erm5'-PstI	AGACTGCAGGAAACGTAAAAGAAGTTATG	Forward primer to amplify erythromycin resistance cassette, used to create pGPI-SceI-XErm, *Pst*I	This study
Erm3'-PstI	GAACTGCAGTACAAATTCCCCGTAGGC	Reverse primer to amplify erythromycin resistance cassette, used to create pGPI-SceI-XErm, *Pst*I	This study
Erm5'-KpnI	AGAGGTACCGAAACGTAAAAGAAGTTATG	Forward primer to amplify erythromycin resistance cassette, used to create pBBR1MCS-6, *Kpn*I	This study
Erm3'-BglII	GAGAGATCTTACAAATTCCCCGTAGGC	Reverse primer to amplify erythromycin resistance cassette, used to create pBBR1MCS-6, *Bgl*II	This study
pBAD18-Up	CCCACTAGTATGTCGGCGATATAG	Forward primer for cloning pBAD promoter and *araC* from pBAD18-Cm into pBBR1MCS-5, *Spe*I	This study
pBAD18-Lw	ATGCTCGAGGGAAATGTTGAATAC	Reverse primer for cloning pBAD promoter and *araC* from pBAD18-Cm into pBBR1MCS-5, *Xho*I	This study
ttg2DcompF	CCAGCTAGCGAGGTTTTCTTCCATGCTG	Forward primer for cloning *ttg2D* (CDS and RBS) into pBBR1-pBAD-Gm, *Nhe*I	This study
ttg2DcompR	TTCAAGCTTGCTGGCCTGGCTCATTTCG	Reverse primer for cloning *ttg2D* (CDS and RBS) into pBBR1-pBAD-Gm, *Hind*III	This study
ttg2DHiscompR	AATAAGCTTTCAGTGGTGGTGGTGGTGGTGTTTCGCCCCGGCCTCTTC	Reverse primer to repetitive histidine codons and for cloning *ttg2D* (CDS and RBS) into pBBR1-pBAD-Gm, *Hind*III	This study
PA4453-Up	ACACCATGGCTCCGACCCCGCAACAG	Forward primer for cloning *ttg2D* (mature protein) into pET28b, *Nco*I	This study
PA4453-Lw	CCTAAGCTTTTTCGCCCCGGCCTCTTC	Reverse primer for cloning *ttg2D* (mature protein) into pET28b, *Hind*III	This study

† Genotype for UW mutants referenced in the following way: gene name-well name as the allele number::Transposon name. tetA, tetracycline-resistance gene. kan, kanamycin-resistance gene. All mutants contained either an ISlacZ/hah or an ISphoA/hah transposon insertion. Between parenthesis strain name at the UW mutant library [http://www.gs.washington.edu/labs/manoil/libraryindex.htm].

‡ MPAO1 (PAO1 for short) was received from the distributor of the PAO1 mutant library of the University of Washington, Seattle [5].

Note: Further information on UW mutants can be found at http://www.gs.washington.edu/labs/manoil/libraryindex.htm. The correct insertion of the transposon into the mutant strains was confirmed in the recent sequence-verified collection of UW mutants [6] and for mutants showing differential phenotypes the transposon location was also confirmed by colony PCR following the protocol and primers recommended by the University of Washington Genome Science Center.

§ The Liverpool epidemic strain (LES) B58, known as LESB58, was kindly donated by Dr. Roger C. Levesque, IBIS, Université Laval, Québec (Canada).

¶ *P. aeruginosa* C17 was isolated from an ICU patient at Hospital Clinic, Barcelona (Spain), upon screening of surveillance rectal swabs in August 2007. *P. aeruginosa* MDR strain PAR-10821 was also isolated at the Hospital Clinic in 2012.

Supplementary Methods

Bacterial growth conditions. Unless stated otherwise, strains were routinely cultured on Luria-Bertani broth (LB) agar plates, or to exponential phase (OD_{550} of 1.0), or up to late exponential phase (OD_{550} of 2.7 to 3.0) in LB at 37°C with shaking at 250 rpm. When necessary, antibiotics were added at final concentrations of 500 µg/ml for erythromycin, 10 µg/ml for gentamicin, 17 µg/ml for tetracycline, 5 µg/ml for norfloxacin, or 50 µg/ml for kanamycin for *Escherichia coli* or 1000 µg/ml for erythromycin, 40 µg/ml for gentamicin, or 60 µg/ml for tetracycline for *Pseudomonas aeruginosa*.

Ttg2D recombinant protein production in *E. coli* and purification. Ttg2D (PA4453) coding sequence from PAO1 strain was cloned from residues 23 (N-terminal) to 215 (C-terminal) followed by a His6 tag into a modified pET28b expression vector (primers listed in Supplementary Table 5). Expression was done in *E. coli* BL21(DE3) in LB broth by inducing with 1 mM IPTG for 3 h. Cells were disrupted by sonication in lysis buffer (0.1% Triton X-100, 4 µg/ml Lysozyme, 8 µg/ml DNase and 2 mM $MgCl_2$), supplemented with a tablet of Protease Inhibitor Cocktail (Roche) per 10 ml of buffer. Recombinant protein in soluble fraction was purified by two chromatographic methods, using an ÄKTA Purifier (GE Healthcare Life sciences). Protein was first purified by metal ion affinity chromatography, using a HisTrap HP 5 ml column (GE Healthcare Life sciences) with the following buffers: binding buffer: 5 mM imidazole, 0.5 M NaCl and 20 mM Tris-HCl (pH 7.9); washing buffer: 40 mM imidazole, 0.33 M NaCl and 13.3 mM Tris-HCl (pH 7.9) and elution buffer: 125 mM imidazole, 62.5 mM NaCl and 2.5 mM Tris-HCl (pH 7.9). Eluted protein was immediately dialyzed against 50 mM Na_2HPO_4 (pH 7.0) to remove the imidazole and then subjected to size-exclusion chromatography using a HiLoad 26/600 Superdex 75 pg column (GE Healthcare Life sciences). Protein concentration was determined by Bradford method and protein purity was evaluated by SDS-PAGE. Purification gave a very good yield, obtaining 99 mg of protein with a purity >99%.

Crystallization, data collection and structure refinement. Purified Ttg2D was concentrated to 18.8 mg/ml in 100 mM NaCl, 10 mM Tris-HCl (pH 7.5) and sitting-drop crystallization trials were performed at 20ºC using commercial screens. Best diffracting crystals were obtained from drops of 200 nl of protein solution plus 200 nl of reservoir solution consisting of 0.17 M ammonium sulfate, 25.5% PEG 4K and 15% glycerol. Harvested crystals were directly flashed cooled in liquid nitrogen. X-ray diffraction data

were collected at 100 K on the beamline ID23-1 at the European Synchrotron Radiation Facility (Grenoble, France) [18]. These data were indexed, integrated, scaled and merged using iMOSFLM [19] and *AIMLESS* [20]. Ttg2D$_{Pae}$ structure was solved with Phaser [21] using a poly-alanine model built with MODELLER [22] from the homologous protein of *Ralstonia solanacearum* (PDB code 2QGU, 25% sequence identity). The structure was automatically re-built with one run of AutoBuild, followed by iterative cycles of restrained refinement with Phenix.refine [23], model building/solvent addition with *Coot* [24] and validation with MolProbity [25]. Geometry restraint information for the phospholipid PG(16:0/cy17:0) was generated from its SMILES description with eLBOW and the semi-empirical quantum mechanical method AM1 [26]. Feature-enhanced map [27] was used to build the lipids as the $2mF_o$ - DF_c electron density was weak in this region. Crystallographic data and refinement statistics are reported in Table 1. Cavities and normal modes were analyzed with the web servers CASTp [28] and ElNémo [29], respectively. The structural alignment was determined with POSA [30] and rendered with ESPript 3 [31] (the PDB sequences were completed to correspond to the UniProt ones). Structural figures were prepared with PyMOL (The PyMOL Molecular Graphics System, Version 1.8 Schrödinger, LLC).

Ttg2D homologous expression in *P. aeruginosa* and purification. A *P. aeruginosa* PAO1 Δ*ttg2D* mutant (Supplementary Table 5) lacking wild-type protein Ttg2D was used for the homologous expression of a His-tagged variant of this protein in its natural environment. The new protein variant was expressed with its own signal peptide and under the control of the arabinose promoter using a modified pBBR1MCS vector. To generate the derivative expression vector were *ttg2*D transcription is controlled by the arabinose promoter (pBAD), first, the pBAD promoter and AraC (arabinose operon regulatory protein) encoding cassette from pBAD18-Cm was cloned into the broad-host-range cloning vector pBBR1MCS-5 (Gm^R) on compatible *Spe*I/*Xho*I sites by using standard techniques and primers pBAD18-Up and pBAD18-Lw (Supplementary Table 5). Then, primers ttg2DcompF and ttg2DHiscompR (Supplementary Table 5) were used to amplify Ttg2D coding sequence including the signal sequence and the RBS, and the resulting amplicon inserted between sites *Nhe*I and *Hind*III into the new vector pBBR1-pBAD-Gm. Primer ttg2DHiscompR includes a sequence coding for six consecutive histidines. The resulting expression vector pBBR1-pBAD-ttg2DHis was introduced into *P. aeruginosa* PAO1 Δ*ttg2D* by electroporation [32].

For protein production, *P. aeruginosa* mutant strain carrying plasmid pBBR1-pBAD-

ttg2DHis was grown in BM2 glycerol medium with casamino acids [62 mM potassium phosphate buffer (pH 7.0), 5 mM $MgSO_4$, 10 µM $FeSO_4$, 0.5% casamino acids and 0.4% glycerol] supplemented with 40 µg/ml gentamicin. A bacterial inoculum from an overnight culture was diluted 1/100 in 1 l of fresh medium and grown to mid-exponential phase (OD_{550} ~ 0.6) at 37°C with vigorous shaking. Cells were centrifuged at 4000 g for 10 min at 4°C, washed twice in 30 mM Tris-HCl (pH 7.0) and 150 mM NaCl and they were kept in ice. The method of spheroplasting by lysozyme and sucrose[33] was applied to extract periplasmic proteins from *P. aeruginosa*. Briefly, the bacterial pellet was resuspended in 30 mM Tris-HCl (pH 8.0), 20% sucrose, 4 mM EDTA, 0.5 mg/ml lysozyme (Sigma-Aldrich, Sigma L6876) and 1 mM PMSF, and incubated for 60 min at 30°C with gentle shaking. After 2 min incubation $MgCl_2$ was added at 10 mM final concentration. The suspension was centrifuged at 11000 g for 15 min at 4°C to collect the supernatant containing periplasmic proteins. The periplasmic protein fraction was filtered through a 0.2 µm sterile filter (Millipore) and immediately dialyzed against 25 mM Tris-HCl (pH 8.0) and 0.3 M NaCl (binding buffer). Protein purification was performed by the ICTS "NANBIOSIS", more specifically the Protein Production Platform of CIBER-BBN/IBB, at the UAB sePBioEs service (Barcelona). The His-tagged protein was purified by metal ion affinity chromatography, using a Ni-NTA affinity column on the ÄKTApure FPLC system (GE Healthcare Life sciences), and eluted with a linear gradient from 0 to 200 mM of Imidazole in binding buffer after being washed extensively with the same buffer. The purified protein was concentrated and desalted in a Centricon micro-concentrator (Millipore).

Native mass spectrometry (Native-MS) analyses. Native-MS experiments were performed using a Synapt G1-HDMS mass spectrometer (Waters, Manchester, UK). All samples were in 100 mM ammonium acetate and were infused by automated chip-based nanoelectrospray using a Triversa Nanomate system (Advion BioSciences, Ithaca, NY, USA) as the interface. The ionization was performed in positive mode using a spray voltage and a gas pressure of 1.75 kV and 0.5 psi, respectively. The source pumping speed in the backing region (6.70 mbar) of the mass spectrometer was reduced to achieve optimal transmission of non-covalent complexes. Cone voltage, extraction cone and source temperature were set to 40 V, 3 V and 40ºC, respectively. Trap and transfer collision energies were set to 6 V and 4 V, respectively. After isolation of the selected ion, fragmentation was performed by CID in the transfer region by applying increasing collision energies (TOF MS/MS analysis). The pressure in the Trap and Transfer T-Wave regions were $5.93 \cdot 10^{-2}$ mbar of Ar and the pressure in the IMS T-Wave was $4.69 \cdot 10^{-1}$ mbar of N_2.

Trap gas flow was 1.5 ml/sec. The bias voltage for entering in the T-wave cell was 15 V. The instrument was calibrated over the m/z range 300-8000 Da using a solution of cesium iodide. MassLynx version 4.1 SCN 704 software was used for data processing. The experimental optimized parameters are listed in detail in the table. Three technical replicates were performed for all MS experiments.

**NanoESI + V Resolution mode**		**DC potentials (V)**	
m/z range	300-8000	Trap Collision Energy	6
Spray voltage (kV)	1.75	Transfer Collision Energy	4
Gas pressure (psi)	0.5	Trap DC Entrance	5
Source Temperature (ºC)	40	Trap DC Bias	15
Sampling Cone (V)	40	Trap DC Exit	5
Extraction Cone (V)	3	IMS DC Entrance	5
Desolvation temperature (ºC)	250	IMS DC Exit	2
Cone Gas Flow (l/h)	10	Transfer DC Entrance	2
Desolvation Gas Flow (l/h)	300	Transfer DC Exit	2
Trap Gas Flow (ml/min)	8		
Backing region (mbar)	6.70		
Trap and Transfer T-Wave section pressure (mbar of Ar)	$5.93 \cdot 10^{-2}$		

Denaturing mass spectrometry. For denaturing MS analysis, samples were directly injected to LTQ-FT Ultra mass spectrometer (Thermo Scientific, USA) using the Triversa Nanomate system. Samples were 1/1 diluted in H_2O/ACN or they were directly injected in ammonium acetate buffer. The NanoMate aspirated the samples from a 384-well plate (protein Lobind) with disposable, conductive pipette tips, and infused the samples through the nanoESI Chip (which consists of 400 nozzles in a 20x20 array) towards the mass spectrometer. Spray voltage was 1.75 kV (positive mode) or -1.80 kV (negative mode) and delivery pressure was 0.50 psi. Capillary temperature, capillary voltage and tube lens were set to 200°C, 35 V (positive ionization) or -37 V (negative ionization), and 100 V, respectively. MS1 and MS2 spectra were acquired at 100 k resolution. Isolated ions were fragmented by CID with collision energy (CE) of 30 eV. Data was acquired with Xcalibur software, vs.2.0SR2 (ThermoScientific). Elemental compositions from experimental exact

mass monoisotopic values were obtained with a dedicated algorithm integrated in Xcalibur software. Three technical replicates were performed for all MS experiments.

For LC-MS (liquid chromatography–mass spectrometry) analysis under denaturing conditions, samples were injected automatically to a BioSuite pPhenyl 1000 (Waters, 10 µm RPC 2.0x75mm) column at a flow rate of 100 µL/min using an Acquity UPLC system (Waters Corporation) provided with a Binary Solvent Manager and an automatic Autosampler. Samples were eluted using a linear gradient from 2% to 5% B in 5min and from 5% to 80% B in 60min (A= 0.1% FA in water, B= 0.1% FA in CH_3CN). The column outlet was directly connected to an LCT-Premier XE mass spectrometer (Waters). Capillary voltage and cone voltage were set to 3000 V and 100 V respectively. Desolvation temperature and source temperature were set to 350ºC and 120ºC. Cone gas flow and desolvation gas flow were set to 50 and 600 L/h. The mass spectrometer acquired full MS scans (400-4000 m/z) working in positive polarity mode. Data was acquired with MassLynx software, V4.1.SCN704 (Waters Inc.). MS spectra corresponding to chromatographic peaks were summed. Charged species in the resulting spectrum were deconvoluted to their zero charged average masses using the integrated MaxEnt1 (maximum entropy) algorithm in MassLynx Software Vs. 4.1.SCN704 (Waters). The algorithm calculates deconvoluted masses peak intensities.

Lipidome analysis from crude cellular extracts. Lipidome analysis of intact cells was done according to Angelini et al.[34] with modifications. Two microliters of lipid extract were mixed with 2 µl of 9-aminoacridine (10 mg/ml dissolved in a 60:40 (v/v) isopropanol:acetonitrile solution) as MALDI matrix and 1 µl of the mixture was spotted on a ground steel plate (Bruker Daltonics, Bremen, Germany). MALDI-MS analyses were performed on an UltrafleXtreme (Bruker Daltonics) and were recorded in the reflectron negative ion mode. The ion acceleration was set to 20 kV. The spectra were processed using Flex Analysis 3.4 software (Bruker Daltonics) and they were analyzed in a mass range between m/z 450 and m/z 1500 Da.

Generation of *ttg2* mutants in MDR *P. aeruginosa* strains using the pGPI-SceI/pDAI-SceI-SacB system. This mutagenesis method is based on the I-SceI homing endonuclease system, which relies on two independent crossover events to integrate first a deletion plasmid with a I-SceI recognition site into the genome of the recipient and then resolve the co-integrate structure by a second homologous recombination event in the

presence of the I-SceI endonuclease provided in trans on a replicative plasmid [13, 35]. One of these mutagenesis systems relies on an improved suicide vector that contains an I-SceI restriction site and the *xylE* reporter gene (pGPI-SceI-XCm), and a replicative but unstable plasmid that encodes the I-SceI endonuclease and the counterselectable marker SacB (pDAI-SceI-SacB) [11]. To successfully achieve genetic manipulations in MDR *P. aeruginosa* strains, we further modified pGPI-SceI-XCm by introducing an erythromycin resistance determinant replacing the chloramphenicol resistance cassette. To make this decision, the MIC for erythromycin was previously analyzed in our *P. aeruginosa* strains using the microdilution technique and found to be at a level of 256 µg/ml. The erythromycin resistance (*erm*) gene from plasmid pNZerm [36] was PCR amplified using primers Erm5'-PstI and Erm3'-PstI (Supplementary Table 5) and with pNZerm DNA as a template. The resulting amplicon (1026 bp) was digested with *Pst*I and cloned into *Pst*I-digested pGPI-SceI-XCm to create pGPI-SceI-XErm. Previously, the region between the *Sac*I sites of plasmid pGPI-SceI-XCm (359 bp) was deleted by digestion with this restriction enzyme and ligation. This region contains the Pc promoter found in class 1 integrons (promoter for the trimethoprim resistance gene *dhfrIIb* in pGPI-SceI vectors) and this could cause unwanted integration of the suicide vector into the *P. aeruginosa* chromosome of some strains. Class 1 integrons have been detected with high prevalence in *P. aeruginosa* [37].

The mutagenesis plasmid for the *ttg2* operon deletion (from PA4452 to PA4456 in PAO1) was constructed by PCR amplification of DNA fragments flanking this gene cluster from PAO1 strain, which were cloned into pGPI-SceI-XErm (see Supplementary Table 5 for primer details). The upstream fragment (633 bp) was amplified using primers US'-ttg2-U and US'-ttg2-L. The downstream fragment (818 bp) was amplified using primers DS'-ttg2-U and DS'-ttg2-L. The upstream fragment was digested with *EcoR*I and *Nhe*I, the downstream fragment was digested with *Nhe*I and *Bgl*II, and both fragments were inserted in two successive cloning steps into pGPI-SceI-XErm to create pΔ*ttg2*-US'DS'. The successful construction of the mutagenesis plasmid was verified by DNA sequence analysis of the inserts. All deletion plasmids were generated and maintained in *E. coli* SY327.

The mutagenic plasmid pΔ*ttg2*-US'DS' was mobilized into *P. aeruginosa* C17, PAER-10821 and LESB58 by triparental mating [35] using *E. coli* DH5α carrying the plasmid pRK2013 as a helper strain. Modifications of the method include that the recipient *P. aeruginosa* strains were incubated at 42°C overnight before conjugation because certain

strains presumably contain restriction systems that could severely restrict foreign DNA. Erythromycin at 1000 µg ml^{-1} was used to select for cointegrants (single-crossover clones) in these *P. aeruginosa* strains, and 5 µg/ml norfloxacin to counter-select against the *E. coli* helper and donor strains. To distinguish true cointegrants from colonies that spontaneously became resistant to erythromycin, streaks of exconjugants were sprayed with 0.45M pyrocatechol since in the presence of this compound colonies expressing 2,3-catechol-dioxygenase encoded by *xylE* turned bright yellow [38]. For the final mutagenesis stage, pDAI-SceI-SacB was mobilized into *P. aeruginosa* cointegrants, and exconjugants were selected on LB agar plates containing 60 µg/ml tetracycline and 5 µg/ml norfloxacin for PAER-10821 and LESB58 derivatives. Tetracycline-resistant colonies, appearing after 48 hours, were screened by PCR and sequencing to confirm the deletion using the primers Ext-ttg2-U and Ext-ttg2-L (Supplementary Table 5) that anneal to sequences outside the deleted region. Detection of deletion mutants cured from the plasmid pDAI-SceI-SacB was achieved by growing *P. aeruginosa* on LB plates without salt and supplemented with 5% (wt/vol) sucrose and then screening the resulting colonies for loss of tetracycline resistance. To obtain a *ttg2* mutant in the *P. aeruginosa* C17 strain, the derivative cointegrates have to be resolved by spontaneous recombination of the allele pair since resolution via the I-SceI endonuclease provided on the plasmid pDAI-SceI-SacB did not work. Failure during the second recombination event for C17 strain (resolution always restores the parental allele) was probably due to the mutation in the *ttg2* operon making the cells more susceptible to tetracycline [39], the antibiotic resistance marker to select for cells carrying plasmid pDAI-SceI-SacB. In this case, resolution of cointegrates was achieved by plating several thousand colonies on LB plates without selection. For this, the single-crossover clones were serially subcultured in LB without selection for two consecutive days and then diluted up to 10^{-8} prior to spread over the plates. Plates were sprayed with 0.45 M pyrocatechol to screen for *xylE* negative cells (no yellow coloration). The screening was facilitated because *P. aeruginosa xylE* negative colonies turned dark brown after spraying with pyrocatechol (Supplementary Fig. 13). Selected colonies were screened by PCR to confirm the deletion using the primers Ext-ttg2-U and Ext-ttg2-L.

Vectors for complementation. For complementation purposes of the PAO1 derivative mutant strains, the broad-host-range cloning vectors pBBR1MCS-5 [14] or its derivative variants pBBR1-pBAD-Gm (see Ttg2D homologous expression in *P. aeruginosa*) or pBBR1MCS-6 were used with the oligonucleotide primers described in Supplementary Table 5 for each cloning strategy. For complementation of the MDR *P. aeruginosa* strains

C17, PAER-10821 and LESB58, we previously modified pBBR1MCS-5 by introducing an erythromycin resistance determinant replacing the gentamicin resistance cassette. The *erm* gene from plasmid pNZerm [36] was PCR amplified using primers Erm5'-KpnI and Erm3'-BglII (Supplementary Table 5) and with pNZerm DNA as a template. The resulting amplicon (1026 bp) was digested with *Kpn*I and *Bgl*II and cloned into *Kpn*I/*Bgl*II -digested pBBR1MCS-5 to create pBBR1MCS-6. The full *ttg2* operon (3650 bp) from *P. aeruginosa* PAO1 was cloned separately into pBBR1MCS-5 or pBBR1MCS-6 as described in Supplementary Table 5. The complementation vectors were introduced into *P. aeruginosa* by electroporation [32].

RNA extraction and real-time quantitative PCR. Early-log-phase cultures of the mutant and complemented strains grown on LB were adjusted to obtain a suspension of approximately 10^9 cell/ml. 0.5 ml of the bacterial suspension were treated with 1 ml of the RNAprotect Reagent (Qiagen) according to the manufacturer's instructions for stabilization of the RNA molecules. Total RNA was extracted by using RNeasy Mini Kit (Qiagen) with on-column DNase digestion (RNase-Free DNase set, Qiagen) to remove contaminating DNA. Removal of DNA was confirmed by performing PCR using an aliquot of the DNase-treated RNA as a template. Reverse transcription reactions were carried out in 20-µl volume containing 0.1 µg RNA, random primers, and the buffer and enzyme components of the Maxima First Strand cDNA Synthesis Kit for RT-qPCR kit (Thermo Fisher Scientific) according to the supplied protocol. Initial PCR amplifications for the expression of *ttg*2 operon-specific mRNAs were performed on the cDNA templates from the parental strains, the Δ*ttg*2 mutants, and the complements to confirm the loss of gene expression in the Δ*ttg*2 deletion mutants and recovery in the complemented strains. Real-time qPCR analysis was carried out on the CFX96 machine at the genomics core facility of Universitat Autònoma de Barcelona using a PCR master mix containing SYBR green dye. The sequences of the *ttg*2D primers used in the real-time qPCR (Int-*ttg*2D-U and Int-*ttg*2D-L) are given in Supplementary Table 5. These primers amplify a 215-bp conserved portion of the *ttg*2D gene. Relative gene expression comparisons were obtained through the $\Delta\Delta C_T$ method (CFX Manager software) by normalizing the mean cycle threshold of the investigated transcript to the housekeeping gene *rpsL* with primers rpsL-F; GCAAGCGCATGGTCGACAAGA) and rpsL-R; CGCTGTGCTCTTGCAGGTTGTGA (amplicon size 201bp).

Outer membrane permeabilization assay. The NPN (1-*N*-phenylnaphthylamine) uptake assay was done according to Loh *et al.* [40], with modifications. Briefly, overnight cultures of the different strains in MHB were subcultured into the same medium and grown to mid-logarithmic phase. Appropriate antibiotic was added to growth plasmid-bearing strains. Cells were washed with 10 mM sodium HEPES (pH 7.2), and then resuspended at a final OD_{550nm} of 1.0 in the same buffer supplemented with a 5 µM CCCP (carbonyl cyanide *m*-chlorophenylhydrazone). CCCP was added to block energized secretion of NPN and to prevent a decline in fluorescence during the assay [40]. 50 µl of cell suspension was pipetted into a quartz cuvette containing NPN (10 µM final concentration), and as test substances either EDTA (0.2 mM final concentration) or colistin (10 µg/ml final concentration) to a total volume of 100 µl. Fluorescence was monitored by a Cari Eclipse spectrophotometer (Variant, Inc., Palo Alto, C.A) at excitation and emission wavelengths of 340 nm and 415 nm, respectively. Control experiments without added cells or without colistin or EDTA were also performed. Each assay was performed at least three times.

Analysis of antimicrobial susceptibilities. Minimal inhibitory concentration (MIC) to antibiotics of several classes including front-line antipseudomonal drugs, namely imipenem, meropenem, amikacin, tobramycin, ceftazidime, piperacillin/tazobactam, ciprofloxacin, levofloxacin and colistin, was determined by the broth microdilution method (BMD) or by Etest. The BMD method was performed on cation adjusted MH broth (CAMHB) as recommended [41, 42]. Bacteria were first grown overnight in CAMHB using CLSI-recommended incubation conditions and the antibiotics were serially diluted twofold across the 96 well plates. After that, 100 µl of a bacterial suspension diluted to $5x10^5$ CFU/ml in CAMHB was added to the wells containing the antibiotic dilutions. The 96-well plates were incubated for 20 h at 37°C before developing by visual inspection or with the resazurin dye [43]. MIC was defined as the lowest concentration of the antibiotic (in µg/ml) that prevented visual growth. As the LESB58 isolate is considered a slower growing strain compared to laboratory strain PAO1 [44], incubation for bacterial growth was extended to 48 h instead of 24 h for some protocols. MICs were confirmed by two or three independent replicates, and MIC values 2-fold or greater than that of the control were considered significant. For Etest a 0.5 McFarland suspension was used to create a confluent lawn of microbial growth in 150-mm Mueller-Hinton (MH) agar plates. The MIC values were determined according to the Etest reading guide after 18 h incubation at 37°C. MICs were determined as the concentration at which the zone of inhibition intersected the Etest strip.

Tolerance to organic solvents, and SDS/EDTA

The approach to assess solvent tolerance involved overlaying solvent (100%) onto LBMg (LB medium supplemented with 10 mM $MgCl_2$) agar plates (55 mm glass plates) inoculated with bacteria as previously described [45]. Briefly, late exponential phase LB broth cultures were diluted into the same medium to yield a suspension of approximately 10^7 cells/ml. A 5-µl aliquot of the cell suspension was spread over the surface of an LBMg agar plate in duplicate and allowed to dry before being overlaid with an organic solvent to a thickness of 3 mm. The plates were sealed and growth was assessed following incubation at 37°C for 24-48 h. Wild-type PAO1 was unable to grow in modified LB agar in the presence of toluene or *n*-hexane, but it grew well in LBMg plates overlaid with *p*-xylene. MIC assays were conducted in 96 well plates to determine the bacterial sensitivity to SDS and/or EDTA. The MIC values were defined as the lowest substance concentration that inhibited 80% of growth (based on OD measurements) in comparison to the growth control.

Biofilm formation. Biofilm formation was assessed as previously described [46] with the following modifications. Sterile 96-well flat bottom polystyrene non-treated plates (BrandTech 781662) were used. Two-hundred microliters of overnight cultures adjusted to an OD_{550nm} of 0.1 were incubated in LB broth or LB supplemented with 0.05 mM EDTA for 24 hours at 37°C. Cells were washed three times with water, fixed at 60°C for 1 h and stained during 15 minutes with 200 µl of 0.1% crystal violet. The dye was discarded and the plate was rinsed in standing water and allowed to dry for 30 min at 37°C. Crystal violet was dissolved in 250 µl 95% ethanol for 15 min, and the OD of the extracted dye was measured at 550 nm. Biofilm formation was normalized by cell growth and reported as relative biofilm formation. A one-way ANOVA with Tukey's multiple comparison test (GraphPad Prism 6.0) was used to determine the significance of the data between groups.

Protein *in-silico* analysis and bioinformatics tools. Protein sequences were analyzed using BLAST, PSI-BLAST and CDD within NCBI (http://ncbi.nlm.nih.gov/) and PsortB (http://psort.nibb.ac.jp). The percentage identity among amino acid sequences was calculated with Clustal Omega (https://www.ebi.ac.uk/Tools/msa/clustalo/). Known 3D structures for *P. aeruginosa* Ttg2D orthologs were downloaded from PDB (https://www.rcsb.org/). Structures with accession numbers 2QGU (*R. solanacearum*) and 4FCZ (*Pseudomonas putida*) were deposited at the PDB by the Northeast Structural Genomics Consortium (NESG) without any associated publications. Structures 5UWA (*E.*

coli) and 5UWB (re-refined coordinates for 4FCZ) were submitted to the PDB by Ekiert *et al*. [1].

Homologous sequences were obtained from a set of representative proteomes at Pfam database (https://pfam.xfam.org/). First, the full sequences of the PF05494 (MlaC family) RP15 group members were downloaded, and Ttg2D orthologs in Gram-negative organisms were then selected from reciprocal best hits. In addition, sequences from unclassified bacteria were removed from the list, totaling 151 representative protein sequences for further analysis. Multiple sequence alignment using a hidden Markov model (HMM) profile was generated with the hmmalign program of the hmmer3 package (http://hmmer.org/), and sequence logos were generated with Weblogo3 [47]. For phylogenetic analysis, an unrooted maximum likelihood tree was reconstructed using the best model of evolution on MEGA 7 [48], based on amino acid sequences. Phylogenetic tree was visualized and annotated using the interactive web platform iTOL v3 [49].

Superposition of the *P. aeruginosa* Ttg2D and 2QGU structures was performed by aligning both sequences to the Pfam HMM profile for the MlaC family with the hmmaling program from the hmmer3 package, and using the resulting alignment to superpose the structures with Profit (http://www.bioinf.org.uk/programs/profit). In order to avoid superposing residues from the C-terminal bended helix, only residues 1 to 175 from Ttg2D and 29 to 199 from 2QGU where used. Superposition of the Ttg2D structure to representative structures of substrate-binding protein subclusters [16] was carried out with the Mammoth program [50]. Structural domains where assigned by transferring those defined for PDB structure 2QGU in CATH database [51]. The search for similar structures in the whole PDB database was performed using Dali server [52].

Supplementary Discussion

Ttg2/Mla pathway and resistance to antimicrobial agents

The proposed function of the Ttg2/Mla pathway in membrane remodeling provides a plausible explanation for the pleiotropic resistance phenotypes shown by the *ttg2* mutants in this study, including resistance to various antibiotics, chelating agents and organic solvents. In addition, these mutations increase the deleterious effect of antibiofilm agents like EDTA, a substance with known low activity against biofilms of *P. aeruginosa* PAO1[53]. Mutations in orthologous *ttg2* genes in other Gram-negative organisms have been shown

to affect diverse physiological processes, mainly associated with an increased OM permeability. In *E. coli*, the mutants defective in components of the Mla system rendered cells more susceptible to the lethal action of quinolones, the detergent SDS and EDTA[54, 55]. Mutants for the orthologs of the Ttg2 pathway in both *Shigella flexneri* and *Francisella novicida* resulted also in increased sensitivity to lysis by SDS[56, 57]. In addition, in *S. flexneri* this pathway appears to play a role in the intercellular spread of the bacteria between adjacent epithelial cells[56]. In fact, the Ttg2/Mla pathway has proven to be an important virulence factor in other pathogens, like *Burkholderia pseudomallei*, that need to spread into neighboring cells to infect eukaryotic tissues[58]. In *Burkholderia cepacia* complex species, *mla* genes are required for swarming motility and serum resistance[59]. Furthermore, in nontypeable *Haemophilus influenzae* (NTHi), it is considered a key factor for bacterial survival in the human airway upon exposure to hydrophobic antibiotics[60]. In *S. flexneri*, *B. pseudomallei* and NTHi the role of the *mla* operon in virulence has been inferred from mutants for the gene *vacJ* (*mlaA*)[58, 61]. This gene is predicted to be part of the Ttg2 ABC transport system, since it is found in an operon with *ttg2* homologs in other bacteria[62]. In agreement with our work, it has been previously shown that in *P. aeruginosa* VacJ plays an important role in both virulence and antibiotic susceptibility to ciprofloxacin, chloramphenicol and tetracycline[63].

Colistin is considered a last-resort antibiotic for the treatment of infections by several MDR Gram-negative pathogens, but its use against MDR *P. aeruginosa* is increasingly impeded by colistin resistance[64]. A variety of gene mutations are known to cause resistance to colistin by altering the OM of Gram-negative bacteria, for example, by covalent modification of the lipid A constituent of LPS as consequence of mutations in the PhoPQ two component regulatory system[65, 66]. In *P. aeruginosa*, the PhoPQ system plays a role in the induction of resistance to polymyxins in response to limiting divalent cations, as well as in virulence[67, 68], and this system has been recently identified as a regulator of *P. aeruginosa*'s *ttg2* operon[39]. Interestingly, in *Salmonella* the increase in OM cardiolipins is regulated by PhoPQ and it is necessary for their virulence[69]. More recently, nucleotide polymorphisms conferring resistance to polymyxins have been detected in genes of the Mla pathway in *A. baumannii*[70]. Although data on the precise mechanisms of resistance are scant and appear to be dependent on specific regulatory systems[67, 71], the activity of the Ttg2 system on membrane phospholipid homeostasis appears to be partly responsible for the lower basal susceptibility of *P. aeruginosa* to colistin.

Supplementary References

1. Ekiert DC*, et al.* Architectures of Lipid Transport Systems for the Bacterial Outer Membrane. *Cell* **169**, 273-285 e217 (2017).

2. Oursel D, Loutelier-Bourhis C, Orange N, Chevalier S, Norris V, Lange CM. Lipid composition of membranes of Escherichia coli by liquid chromatography/tandem mass spectrometry using negative electrospray ionization. *Rapid Commun Mass Sp* **21**, 1721-1728 (2007).

3. Gidden J, Denson J, Liyanage R, Ivey DM, Lay JO. Lipid compositions in Escherichia coli and Bacillus subtilis during growth as determined by MALDI-TOF and TOF/TOF mass spectrometry. *Int J Mass Spectrom* **283**, 178-184 (2009).

4. Groenewold MK*, et al.* A phosphatidic acid-binding protein is important for lipid homeostasis and adaptation to anaerobic biofilm conditions in Pseudomonas aeruginosa. *Biochem J* **475**, 1885-1907 (2018).

5. Jacobs MA*, et al.* Comprehensive transposon mutant library of Pseudomonas aeruginosa. *Proc Natl Acad Sci U S A* **100**, 14339-14344 (2003).

6. Held K, Ramage E, Jacobs M, Gallagher L, Manoil C. Sequence-verified two-allele transposon mutant library for Pseudomonas aeruginosa PAO1. *J Bacteriol* **194**, 6387-6389 (2012).

7. Cheng K*, et al.* Spread of beta-lactam-resistant Pseudomonas aeruginosa in a cystic fibrosis clinic. *Lancet* **348**, 639-642 (1996).

8. Smart CH, Walshaw MJ, Hart CA, Winstanley C. Use of suppression subtractive hybridization to examine the accessory genome of the Liverpool cystic fibrosis epidemic strain of Pseudomonas aeruginosa. *J Med Microbiol* **55**, 677-688 (2006).

9. Hanahan D. Studies on Transformation of Escherichia-Coli with Plasmids. *Journal of Molecular Biology* **166**, 557-580 (1983).

10. Miller VL, Mekalanos JJ. A Novel Suicide Vector and Its Use in Construction of Insertion Mutations - Osmoregulation of Outer-Membrane Proteins and Virulence Determinants in Vibrio-Cholerae Requires Toxr. *Journal of Bacteriology* **170**, 2575-2583 (1988).

11. Hamad MA, Skeldon AM, Valvano MA. Construction of Aminoglycoside-Sensitive Burkholderia cenocepacia Strains for Use in Studies of Intracellular Bacteria with the Gentamicin Protection Assay. *Appl Environ Microb* **76**, 3170-3176 (2010).

12. Figurski DH, Helinski DR. Replication of an Origin-Containing Derivative of Plasmid Rk2 Dependent on a Plasmid Function Provided in Trans. *P Natl Acad Sci USA* **76**, 1648-1652 (1979).

13. Flannagan RS, Linn T, Valvano MA. A system for the construction of targeted unmarked gene deletions in the genus Burkholderia. *Environmental Microbiology* **10**, 1652-1660 (2008).

14. Kovach ME*, et al.* Four new derivatives of the broad-host-range cloning vector pBBR1MCS, carrying different antibiotic-resistance cassettes. *Gene* **166**, 175-176 (1995).

15. Guzman LM, Belin D, Carson MJ, Beckwith J. Tight regulation, modulation, and high-level expression by vectors containing the arabinose PBAD promoter. *J Bacteriol* **177**, 4121-4130 (1995).

16. Scheepers GH, Lycklama A Nijeholt JA, Poolman B. An updated structural classification of substrate-binding proteins. *FEBS letters* **590**, 4393-4401 (2016).

17. Zhou Y, Peisker H, Dormann P. Molecular species composition of plant cardiolipin determined by liquid chromatography mass spectrometry. *J Lipid Res* **57**, 1308-1321 (2016).

18. Nurizzo D*, et al.* The ID23-1 structural biology beamline at the ESRF. *Journal of Synchrotron Radiation* **13**, 227-238 (2006).

19. Battye TGG, Kontogiannis L, Johnson O, Powell HR, Leslie AGW. iMOSFLM: a new graphical interface for diffraction-image processing with MOSFLM. *Acta Crystallographica Section D: Biological Crystallography* **67**, 271-281 (2011).

20. Evans PR, Murshudov GN. How good are my data and what is the resolution? *Acta Crystallographica Section D: Biological Crystallography* **69**, 1204-1214 (2013).

21. McCoy AJ, Grosse-Kunstleve RW, Adams PD, Winn MD, Storoni LC, Read RJ. Phaser crystallographic software. *Journal of Applied Crystallography* **40**, 658-674 (2007).

22. Eswar N*, et al.* Comparative Protein Structure Modeling Using Modeller. *Current protocols in bioinformatics / editoral board, Andreas D Baxevanis [et al]* **0 5**, Unit-5.6 (2006).

23. Adams PD*, et al.* PHENIX: a comprehensive Python-based system for macromolecular structure solution. *Acta Crystallogr D Biol Crystallogr* **66**, 213-221 (2010).

24. Emsley P, Lohkamp B, Scott WG, Cowtan K. Features and development of Coot. *Acta Crystallographica Section D: Biological Crystallography* **66**, 486-501 (2010).

25. Chen VB*, et al.* MolProbity: all-atom structure validation for macromolecular crystallography. *Acta Crystallographica Section D: Biological Crystallography* **66**, 12-21 (2010).

26. Moriarty NW, Grosse-Kunstleve RW, Adams PD. electronic Ligand Builder and Optimization Workbench (eLBOW): a tool for ligand coordinate and restraint generation. *Acta Crystallogr D Biol Crystallogr* **65**, 1074-1080 (2009).

27. Afonine PV*, et al.* FEM: feature-enhanced map. *Acta Crystallogr D Biol Crystallogr* **71**, 646-666 (2015).

28. Dundas J, Ouyang Z, Tseng J, Binkowski A, Turpaz Y, Liang J. CASTp: computed atlas of surface topography of proteins with structural and topographical mapping of functionally annotated residues. *Nucleic Acids Res* **34**, W116-W118 (2006).

29. Suhre K, Sanejouand Y-H. ElNémo: a normal mode web server for protein movement analysis and the generation of templates for molecular replacement. *Nucleic Acids Res* **32**, W610-W614 (2004).

30. Li Z, Natarajan P, Ye Y, Hrabe T, Godzik A. POSA: a user-driven, interactive multiple protein structure alignment server. *Nucleic Acids Res* **42**, W240-W245 (2014).

31. Robert X, Gouet P. Deciphering key features in protein structures with the new ENDscript server. *Nucleic Acids Res* **42**, W320-W324 (2014).

32. Choi KH, Kumar A, Schweizer HP. A 10-min method for preparation of highly electrocompetent Pseudomonas aeruginosa cells: application for DNA fragment transfer between chromosomes and plasmid transformation. *J Microbiol Methods* **64**, 391-397 (2006).

33. Imperi F*, et al.* Analysis of the periplasmic proteome of Pseudomonas aeruginosa, a metabolically versatile opportunistic pathogen. *Proteomics* **9**, 1901-1915 (2009).

34. Angelini R*, et al.* Lipidomics of intact mitochondria by MALDI-TOF/MS. *J Lipid Res* **53**, 1417-1425 (2012).

35. Aubert DF, Hamad MA, Valvano MA. A Markerless Deletion Method for Genetic Manipulation of Burkholderia cenocepacia and Other Multidrug-Resistant Gram-Negative Bacteria. *Host-Bacteria Interactions: Methods and Protocols* **1197**, 311-327 (2014).

36. Podbielski A, Peterson JA, Cleary P. Surface Protein-Cat Reporter Fusions Demonstrate Differential Gene-Expression in the Vir Regulon of Streptococcus-Pyogenes. *Molecular Microbiology* **6**, 2253-2265 (1992).

37. Deng Y*, et al.* Resistance integrons: class 1, 2 and 3 integrons. *Ann Clin Microb Anti* **14**, (2015).

38. Hamad MA, Zajdowicz SL, Holmes RK, Voskuil MI. An allelic exchange system for compliant genetic manipulation of the select agents Burkholderia pseudomallei and Burkholderia mallei. *Gene* **430**, 123-131 (2009).

39. Chen L, Duan KM. A PhoPQ-Regulated ABC Transporter System Exports Tetracycline in Pseudomonas aeruginosa. *Antimicrob Agents Ch* **60**, 3016-3024 (2016).

40. Loh B, Grant C, Hancock RE. Use of the fluorescent probe 1-N-phenylnaphthylamine to study the interactions of aminoglycoside antibiotics with the outer membrane of Pseudomonas aeruginosa. *Antimicrob Agents Chemother* **26**, 546-551 (1984).

41. CLSI. Performance standards for antimicrobial susceptibility testing; 27th edition. CLSI M100-S27. Clinical and Laboratory Standards Institute, Wayne, PA., (2017).

42. CLSI. Methods for dilution antimicrobial susceptibility tests for bacteria that grow aerobically; approved standard. Ninth Edition. CLSI M07-A9. Clinical and Laboratory Standards Institute, Wayne, PA., (2012).

43. Sarker SD, Nahar L, Kumarasamy Y. Microtitre plate-based antibacterial assay incorporating resazurin as an indicator of cell growth, and its application in the in vitro antibacterial screening of phytochemicals. *Methods* **42**, 321-324 (2007).

44. Jeukens J*, et al.* Comparative Genomics of Isolates of a Pseudomonas aeruginosa Epidemic Strain Associated with Chronic Lung Infections of Cystic Fibrosis Patients. *PLoS One* **9**, e87611 (2014).

45. Li XZ, Zhang L, Poole K. Role of the multidrug efflux systems of Pseudomonas aeruginosa in organic solvent tolerance. *J Bacteriol* **180**, 2987-2991 (1998).

46. O'Toole GA, Pratt LA, Watnick PI, Newman DK, Weaver VB, Kolter R. Genetic approaches to study of biofilms. *Method Enzymol* **310**, 91-109 (1999).

47. Crooks GE, Hon G, Chandonia JM, Brenner SE. WebLogo: a sequence logo generator. *Genome Res* **14**, 1188-1190 (2004).

48. Kumar S, Stecher G, Tamura K. MEGA7: Molecular Evolutionary Genetics Analysis Version 7.0 for Bigger Datasets. *Mol Biol Evol* **33**, 1870-1874 (2016).

49. Letunic I, Bork P. Interactive tree of life (iTOL) v3: an online tool for the display and annotation of phylogenetic and other trees. *Nucleic Acids Res* **44**, W242-245 (2016).

50. Ortiz AR, Strauss CE, Olmea O. MAMMOTH (matching molecular models obtained from theory): an automated method for model comparison. *Protein Sci* **11**, 2606-2621 (2002).

51. Sillitoe I*, et al.* CATH: comprehensive structural and functional annotations for genome sequences. *Nucleic Acids Research* **43**, D376-D381 (2015).

52. Holm L, Rosenstrom P. Dali server: conservation mapping in 3D. *Nucleic Acids Research* **38**, W545-W549 (2010).

53. Banin E, Brady KM, Greenberg EP. Chelator-induced dispersal and killing of Pseudomonas aeruginosa cells in a biofilm. *Appl Environ Microbiol* **72**, 2064-2069 (2006).

54. Han X, Geng J, Zhang L, Lu T. The role of Escherichia coli YrbB in the lethal action of quinolones. *J Antimicrob Chemother* **66**, 323-331 (2011).

55. Malinverni JC, Silhavy TJ. An ABC transport system that maintains lipid asymmetry in the gram-negative outer membrane. *Proc Natl Acad Sci U S A* **106**, 8009-8014 (2009).

56. Carpenter CD*, et al.* The Vps/VacJ ABC transporter is required for intercellular spread of Shigella flexneri. *Infect Immun* **82**, 660-669 (2014).

57. Enstrom M, Held K, Ramage B, Brittnacher M, Gallagher L, Manoil C. Genotype-phenotype associations in a nonmodel prokaryote. *MBio* **3**, (2012).

58. Cuccui J, *et al.* Development of signature-tagged mutagenesis in Burkholderia pseudomallei to identify genes important in survival and pathogenesis. *Infect Immun* **75**, 1186-1195 (2007).

59. Bernier SP, Son S, Surette MG. The Mla Pathway Plays an Essential Role in the Intrinsic Resistance of Burkholderia cepacia Complex Species to Antimicrobials and Host Innate Components. *J Bacteriol* **200**, (2018).

60. Fernandez-Calvet A, *et al.* Modulation of Haemophilus influenzae interaction with hydrophobic molecules by the VacJ/MlaA lipoprotein impacts strongly on its interplay with the airways. *Sci Rep* **8**, 6872 (2018).

61. Suzuki T, Murai T, Fukuda I, Tobe T, Yoshikawa M, Sasakawa C. Identification and characterization of a chromosomal virulence gene, vacJ, required for intercellular spreading of Shigella flexneri. *Mol Microbiol* **11**, 31-41 (1994).

62. Roier S, *et al.* A novel mechanism for the biogenesis of outer membrane vesicles in Gram-negative bacteria. *Nat Commun* **7**, 10515 (2016).

63. Shen L, *et al.* PA2800 plays an important role in both antibiotic susceptibility and virulence in Pseudomonas aeruginosa. *Curr Microbiol* **65**, 601-609 (2012).

64. Lim LM, *et al.* Resurgence of colistin: a review of resistance, toxicity, pharmacodynamics, and dosing. *Pharmacotherapy* **30**, 1279-1291 (2010).

65. Miller AK, *et al.* PhoQ mutations promote lipid A modification and polymyxin resistance of Pseudomonas aeruginosa found in colistin-treated cystic fibrosis patients. *Antimicrob Agents Chemother* **55**, 5761-5769 (2011).

66. Barrow K, Kwon DH. Alterations in two-component regulatory systems of phoPQ and pmrAB are associated with polymyxin B resistance in clinical isolates of Pseudomonas aeruginosa. *Antimicrob Agents Chemother* **53**, 5150-5154 (2009).

67. Macfarlane EL, Kwasnicka A, Hancock RE. Role of Pseudomonas aeruginosa PhoP-phoQ in resistance to antimicrobial cationic peptides and aminoglycosides. *Microbiology* **146 (Pt 10)**, 2543-2554 (2000).

68. Gooderham WJ, *et al.* The sensor kinase PhoQ mediates virulence in Pseudomonas aeruginosa. *Microbiology* **155**, 699-711 (2009).

69. Dalebroux ZD, *et al.* Delivery of cardiolipins to the Salmonella outer membrane is necessary for survival within host tissues and virulence. *Cell Host Microbe* **17**, 441-451 (2015).

70. Powers MJ, Trent MS. Phospholipid retention in the absence of asymmetry strengthens the outer membrane permeability barrier to last-resort antibiotics. *Proc Natl Acad Sci U S A* **115**, E8518-E8527 (2018).

71. Moskowitz SM, *et al.* PmrB mutations promote polymyxin resistance of Pseudomonas aeruginosa isolated from colistin-treated cystic fibrosis patients. *Antimicrob Agents Chemother* **56**, 1019-1030 (2012).
