# Supplementary material for: The Pseudomonas aeruginosa substrate-binding protein Ttg2D functions as a general glycerophospholipid transporter across the periplasm
Source: Commun Biol. 2021 Apr 9;4:448. doi: 10.1038/s42003-021-01968-8 (PMC8035174; doi:10.1038/s42003-021-01968-8)
Supplement: Supplementary file 3 — Description of Additional Supplementary Files [file 42003_2021_1968_MOESM3_ESM.pdf]

Description of Additional Supplementary Files

File name: Supplementary Data 1

Description: Source Data.
