# Supplementary material for: The Pseudomonas aeruginosa substrate-binding protein Ttg2D functions as a general glycerophospholipid transporter across the periplasm
Source: Commun Biol. 2021 Apr 9;4:448. doi: 10.1038/s42003-021-01968-8 (PMC8035174; doi:10.1038/s42003-021-01968-8)
Supplement: Supplementary file 5 — Reporting Summary [file 42003_2021_1968_MOESM5_ESM.pdf]

nature research

Corresponding author(s): Xavier Daura

Last updated by author(s): Feb 15, 2021

Reporting Summary

Nature Research wishes to improve the reproducibility of the work that we publish. This form provides structure for consistency and transparency in reporting. For further information on Nature Research policies, see our Editorial Policies and the Editorial Policy Checklist.

Statistics

For all statistical analyses, confirm that the following items are present in the figure legend, table legend, main text, or Methods section.

n/a	Confirmed	
☐	☒	The exact sample size (n) for each experimental group/condition, given as a discrete number and unit of measurement
☐	☒	A statement on whether measurements were taken from distinct samples or whether the same sample was measured repeatedly
☐	☒	The statistical test(s) used AND whether they are one- or two-sided<br>*Only common tests should be described solely by name; describe more complex techniques in the Methods section.*
☒	☐	A description of all covariates tested
☒	☐	A description of any assumptions or corrections, such as tests of normality and adjustment for multiple comparisons
☐	☒	A full description of the statistical parameters including central tendency (e.g. means) or other basic estimates (e.g. regression coefficient) AND variation (e.g. standard deviation) or associated estimates of uncertainty (e.g. confidence intervals)
☒	☐	For null hypothesis testing, the test statistic (e.g. F, t, r) with confidence intervals, effect sizes, degrees of freedom and P value noted<br>*Give P values as exact values whenever suitable.*
☒	☐	For Bayesian analysis, information on the choice of priors and Markov chain Monte Carlo settings
☒	☐	For hierarchical and complex designs, identification of the appropriate level for tests and full reporting of outcomes
☒	☐	Estimates of effect sizes (e.g. Cohen's d, Pearson's r), indicating how they were calculated

Our web collection on statistics for biologists contains articles on many of the points above.

Software and code

Policy information about availability of computer code

Data collection	X-ray diffraction data were collected on the beamline ID23-1 at the European Synchrotron Radiation Facility (Grenoble, France). Triversa Nanomate system (Advion BioSciences, Ithaca, NY, USA) coupled to Synapt G1-HDMS mass spectrometer (Waters, Manchester, UK) or to LTQ-FT Ultra mass spectrometer (Thermo Scientific, USA) and an LCT-Premier XE mass spectrometer (Waters) were used to collect the MS data for native and denaturing MS experiments. Data was acquired with Xcalibur software, vs.2.0SR2 (ThermoScientific) or MassLynx software V4.1.SCN704 (Waters Inc.). MALDI-MS analyses were performed on an UltrafleXtreme (Bruker Daltonics). Collection of similar structures in the PDB database using Dali server.
Data analysis	Flex Analysis 3.4 software (Bruker Daltonics), MassLynx version 4.1 SCN 704 and Drift scope version 2.4 software were used for MS data processing. CFX Manager software, iMOSFLM 2 and AIMLESS, MODELLER, Phaser, Phenix.refine, Coot, MolProbity, eLBOW, PyMOL 1.8, web servers CASTp and ElNémo, POSA, ESPript 3, hmmalign and Mammoth programs, Clustal Omega, Weblogo3, MEGA 7, Graphpad prism 6.

For manuscripts utilizing custom algorithms or software that are central to the research but not yet described in published literature, software must be made available to editors and reviewers. We strongly encourage code deposition in a community repository (e.g. GitHub). See the Nature Research guidelines for submitting code & software for further information.

Data

Policy information about availability of data

All manuscripts must include a data availability statement. This statement should provide the following information, where applicable:

- Accession codes, unique identifiers, or web links for publicly available datasets
- A list of figures that have associated raw data
- A description of any restrictions on data availability

Data that support the findings of this study have been deposited in RSCB Protein Data Bank with the accession code 6HSY. New plasmid sequences have been

deposited in GenBank (NCBI) with identifiers KY368390 and KY368389. All other data that support the findings of this study are available from the corresponding author upon reasonable request. Source data are available in the source data file for Figs. 2, 5 and 6 and supplementary figures S7 and S13.

Field-specific reporting

Please select the one below that is the best fit for your research. If you are not sure, read the appropriate sections before making your selection.

☒ Life sciences ☐ Behavioural & social sciences ☐ Ecological, evolutionary & environmental sciences

For a reference copy of the document with all sections, see nature.com/documents/nr-reporting-summary-flat.pdf

Life sciences study design

All studies must disclose on these points even when the disclosure is negative.

Sample size	Owing to the nature of our investigations no sample size calculations were performe.
Data exclusions	Data is not excluded.
Replication	All attempts at replication were successful and we confirmed it.
Randomization	No groups are present in this study.
Blinding	Owing to the nature of our investigations blinding was not feasible.

Reporting for specific materials, systems and methods

We require information from authors about some types of materials, experimental systems and methods used in many studies. Here, indicate whether each material, system or method listed is relevant to your study. If you are not sure if a list item applies to your research, read the appropriate section before selecting a response.

Materials & experimental systems

n/a	Involved in the study	
☒	☐	Antibodies
☒	☐	Eukaryotic cell lines
☒	☐	Palaeontology and archaeology
☒	☐	Animals and other organisms
☒	☐	Human research participants
☒	☐	Clinical data
☒	☐	Dual use research of concern

Methods

n/a	Involved in the study	
☒	☐	ChIP-seq
☒	☐	Flow cytometry
☒	☐	MRI-based neuroimaging
